# A new approach to ferrocene derived alkenes via copper-catalyzed olefination

**DOI:** 10.3762/bjoc.11.223

**Published:** 2015-11-03

**Authors:** Vasily M Muzalevskiy, Aleksei V Shastin, Alexandra D Demidovich, Namiq G Shikhaliev, Abel M Magerramov, Victor N Khrustalev, Rustem D Rakhimov, Sergey Z Vatsadze, Valentine G Nenajdenko

**Affiliations:** 1 Department of Chemistry, Lomonosov Moscow State University, Moscow 119991, Russia; 2 Institute of Problems of Chemical Physics, Russian Academy of Sciences, Chernogolovka, Moscow region, 142432 Russia; 3 Baku State University, Department of Chemistry, Z. Xalilov Str. 23, Az 1148 Baku, Azerbaijan; 4 Peoples’ Friendship University of Russia; 5 A.N. Nesmeyanov Institute of Organoelement Compounds, Russian Academy of Sciences, 28 Vavilov Street, Moscow 119991, Russia

**Keywords:** catalytic olefination, copper catalysis, cyclic voltammetry, ferrocene

## Abstract

A new approach to ferrocenyl haloalkenes and bis-alkenes was elaborated. The key procedure involves copper catalyzed olefination of *N*-unsubstituted hydrazones, obtained from ferrocene-containing carbonyl compounds and hydrazine, with polyhaloalkanes. The procedure is simple, cheap and could be applied for the utilization of environmentally harmful polyhalocarbons. The cyclic voltammetry study of the representative examples of the synthesized ferrocenyl alkenes shows the strong dependence of the cathodic behavior on the amount of vinyl groups: while for the monoalkene containing molecules no reduction is seen, the divinyl products are reduced in several steps.

## Introduction

**Scheme 1:**
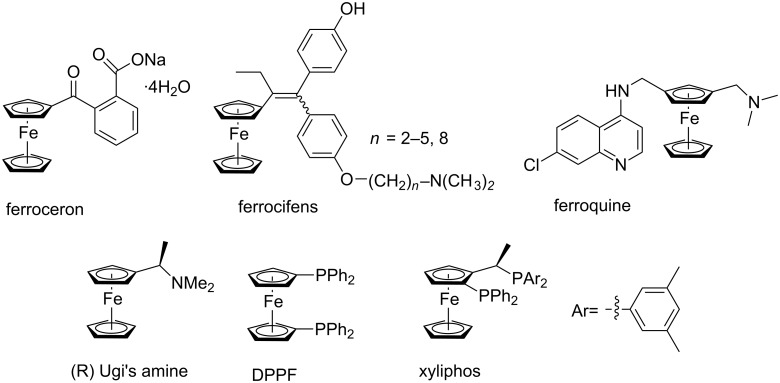
Examples of ferrocene derived drugs and ligands.

The introduction of complex functional fragments into the specified place of the target molecule is of current interest in modern synthetic chemistry [1]. From this point of view, the development of ferrocene-based molecules as crucial fragments of new materials and novel pharmacological entities is of great importance. Indeed, since the discovery of ferrocene [2] and the synthesis of the first polymer based on vinylferrocene [3], the chemistry of ferrocene and its derivatives is developing very rapidly. Ferrocene derivatives are widely applied in industry, for example, diethylferrocene is a combustion accelerator used as additive to gasoline [4]. There are many drugs containing a ferrocene fragment in their structures (Scheme 1) [5]. The ferrocene core is also a very popular scaffold for ligand design, particularly, in asymmetric catalysis (Scheme 1).

**Scheme 2:**
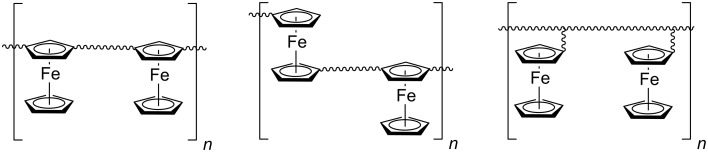
Structural types of ferrocene-based polymers.

Probably, the most challenging application of ferrocenes is the production of ferrocene-containing polymers [6–8] (Scheme 2). The polymers have a set of unique properties and are used in various fields of science, technology and medicine (conducting and semiconducting materials [9], drugs [10], biosensors [11–13], liquid crystal materials [14–15], coordination polymers [16], and more [10]).

Ethynylferrocenes are one of the most popular and extremely effective starting compounds in the creation of ferrocenyl polymers. They are widely used for making both polyferrocenylvinylenes (double bond linkers) [17–21] and polyethynylferrocenes (triple bond linkers) [22–25]. There are several methods for the preparation of ethynylferrocenes [24–29]. In most cases, the key reagents for the synthesis of ethynylferrocene are the corresponding mono- and dihalogenvinylferrocene derivatives. These compounds are generally prepared using a variation of the Wittig reaction (the Corey–Fuchs reaction) using a 2–4-fold molar excess of triphenylphosphine [27–31].

## Results and Discussion

### Synthesis of halovinylferrocenes

A few years ago we discovered a new reaction for a double carbon–carbon bond formation – the reaction of catalytic olefination. It was shown that the copper-catalyzed reaction of unsubstituted hydrazones of aromatic (aliphatic) aldehydes and ketones with a wide range of polyhalogenalkanes leads to the corresponding substituted ethylenes with one or two geminal halogen atoms [32–36]. In the present study, we investigated the possibility of using a catalytic olefination reaction for the synthesis of ferrocene derivatives. As a result, several well-known and previously unknown ferrocene-containing alkene compounds were obtained.

**Scheme 3:**
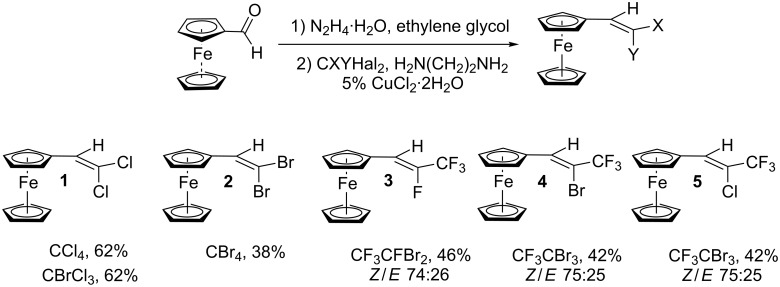
Synthesis of ferrocene-derived alkenes from ferrocene carbaldehyde.

First, ferrocene carbaldehyde was investigated. It was found, that under usual conditions of the reaction (equal amount of N_2_H_4_·H_2_O, DMSO or EtOH as a solvent) the yields of the desired alkenes were not good enough. Better results were obtained in ethylene glycol with a 4-fold excess of N_2_H_4_·H_2_O (novel media just reported for COR [37]). In this case, target alkenes were isolated in up to 62% yield. Using C2-freons fluorinated alkenes **3–5** were synthesized (Scheme 3). The reaction proceeds stereoselectively to give a mixture of isomers in which the less hindered *Z*-isomer dominates. The assignment of the isomers was easily performed by comparison of NMR spectral data with those previously reported for the *Z,E*-isomers of similar alkenes [38–40]. The trifluoromethyl group of the *Z*-isomers of compounds **3**–**5** resonates in higher field in ^19^F NMR (**3**: −72.8 ppm, **4**: −67.1 ppm, **5**: −69.0 ppm) than that of the *E*-isomers (**3**: −67.9 ppm, **4**: −60.2 ppm, **5**: −62.4 ppm). In the case of compound **3** an additional conformation can be seen in the ^1^H NMR spectrum. It is well-known, that ^2^*J*_HF_ coupling constants in alkenes with relative *trans*-configuration of hydrogen and fluorine atoms are usually approximately twice bigger than the corresponding coupling constants in alkenes with *cis*-configuration. The values found in **3** are 36.5 Hz (*Z*-isomer by systematic nomenclature, but *trans*-configuration of H and F) and 16.2 Hz (*E*-isomer).

**Scheme 4:**
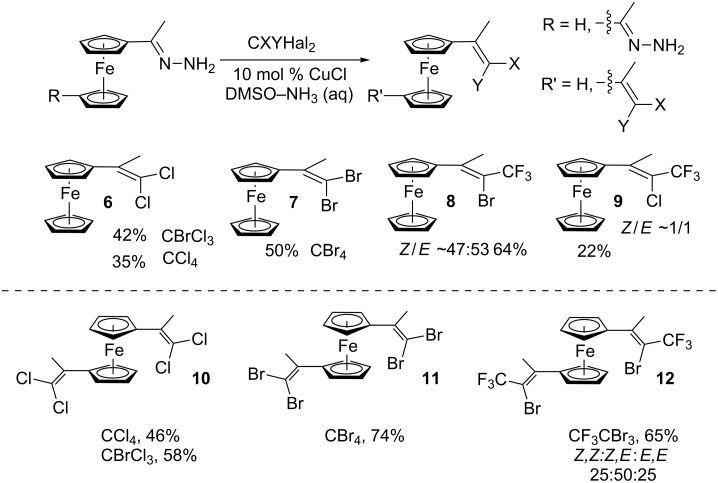
Synthesis of ferrocene-derived alkenes from acetylferrocene and 1,1’-diacetylferrocene.

Acetylferrocene and 1,1’-diacetylferrocene were also involved successfully into this transformation (Scheme 4). Our preliminary study of the catalytic olefination of acetylferrocene by CF_3_CBr_3_ (alkene **8**) and CBr_4_ (alkene **7**) indicates, that in contrast to ferrocene carbaldehyde, better results were achieved in DMSO using previously prepared hydrazones [37]. A series of ferrocene derivatives including fluorinated ones was prepared in good yields. Unsymmetrical alkenes **8** and **9** were obtained as a mixture of isomers in approximately equal amounts. Identification of the structures of the *E-* and *Z*-isomers of alkene **8** was accurately performed by an X-ray crystallographic study using crystals of **8** obtained from ethanol solution (see Supporting Information File 1 and [41]). Due to the presence of two double bonds in alkene **12** the formation of three isomers (*Z,Z*, *Z,E* and *E,E*) is possible. Similar to alkenes **8** and **9** no stereoselectivity is observed to give equal numbers of *cis*- and *trans*-double bonds in the obtained molecules. As a result a 25:50:25 mixture of *Z,Z*, *Z,E* and *E,E* was formed. The higher quantity of the *Z,E*-isomer is a result of statistical doubling (in fact, the reaction gives *Z,E* and *E,Z*-alkenes which are identical).

Di- and tetrahalovinylferrocenes obtained in our work are of interest for the synthesis of ethynylferrocenes, as monomers for making ferrocene-containing polymers and as intermediates in the synthesis of ferrocene analogs of tamoxifen and other medicinally relevant molecules.

### Electrochemical properties of halovinylferrocenes

The ferrocene unit possesses several exciting electrochemical characteristics, such as fast electron-transfer rate, low oxidation potential, and stability of two redox states. The combination of a ferrocene and an alkene moiety in one molecule might be of interest for the synthesis of functional devises that can be exploited in electrocatalysis, electroanalysis, and biosensing applications, since the alkene fragment could be used to graft the molecule to the polymer support or to copolymerize it with the appropriate monomers. Therefore, we have also examined the electrochemical properties of the compounds obtained. It was shown that the oxidation of the ferrocene unit proceeds in accordance to the data found in literature, whereas the reduction was found only in the case of bis(halovinyl)ferrocenes. Our results clearly indicate that the anodic and cathodic electrochemical processes proceed on different parts of the molecules. While in the anodic region the changes are localized on the iron atom, the electron transfer from the cathode occurs at a double bond.

Three representative groups of halovinylferrocenes were studied using cyclic voltammetry (Table 1). The first group was formed from three omega-dichloro derivatives **1**, **6**, and **10**. The second one was made from three omega-dibromo products **2**, **7**, and **11**, and the last group consisted of bromotrifluoromethyl molecules **4**, **8**, and **12**. Such selection allowed us to estimate the effect of the nature of substituent on the electrochemical behavior of the molecule.

**Table 1:** Cyclic voltammetry data for selected halovinylferrocenes. Pt electrode, DMF, Ag/AgCl/KCl (sat.), Bu_4_NBF_4_.

Compound	*E*_p_^Ox^, V	−*E*_p_^Red1^, V	−*E*_p_^Red2^, V	−*E*_p_^Red3^, V

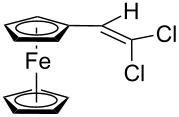 **1**	0.646 (0.580)^a^	–	–	–
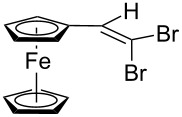 **2**	0.651 (0.584)	–	–	–
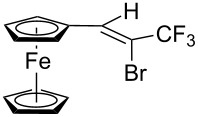 **4**	0.735 (0.669)	–	–	–
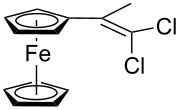 **6**	0.655 (0.576)		–	–
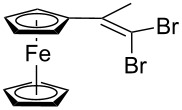 **7**	0.641 (0.579)	–	–	–
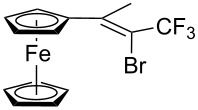 **8**	0.726 (0.607)	–	–	–
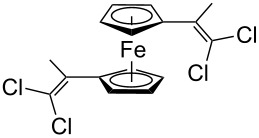 **10**	0.741 (0.675)	0.371 IR^b^	0.983 (0.763)	–
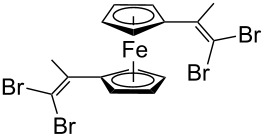 **11**	0.740 (0.670)	0.68 IR	1.022 IR	1.66 IR
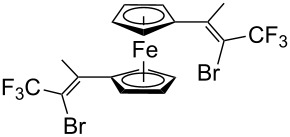 **12**	0.836 (0.867)	0.343 IR	1.232 (0.71)	–

^a^In the case of a reversible peak the reverse potentials are shown in paranthesis. ^b^IR means irreversible.

**Figure 1:**
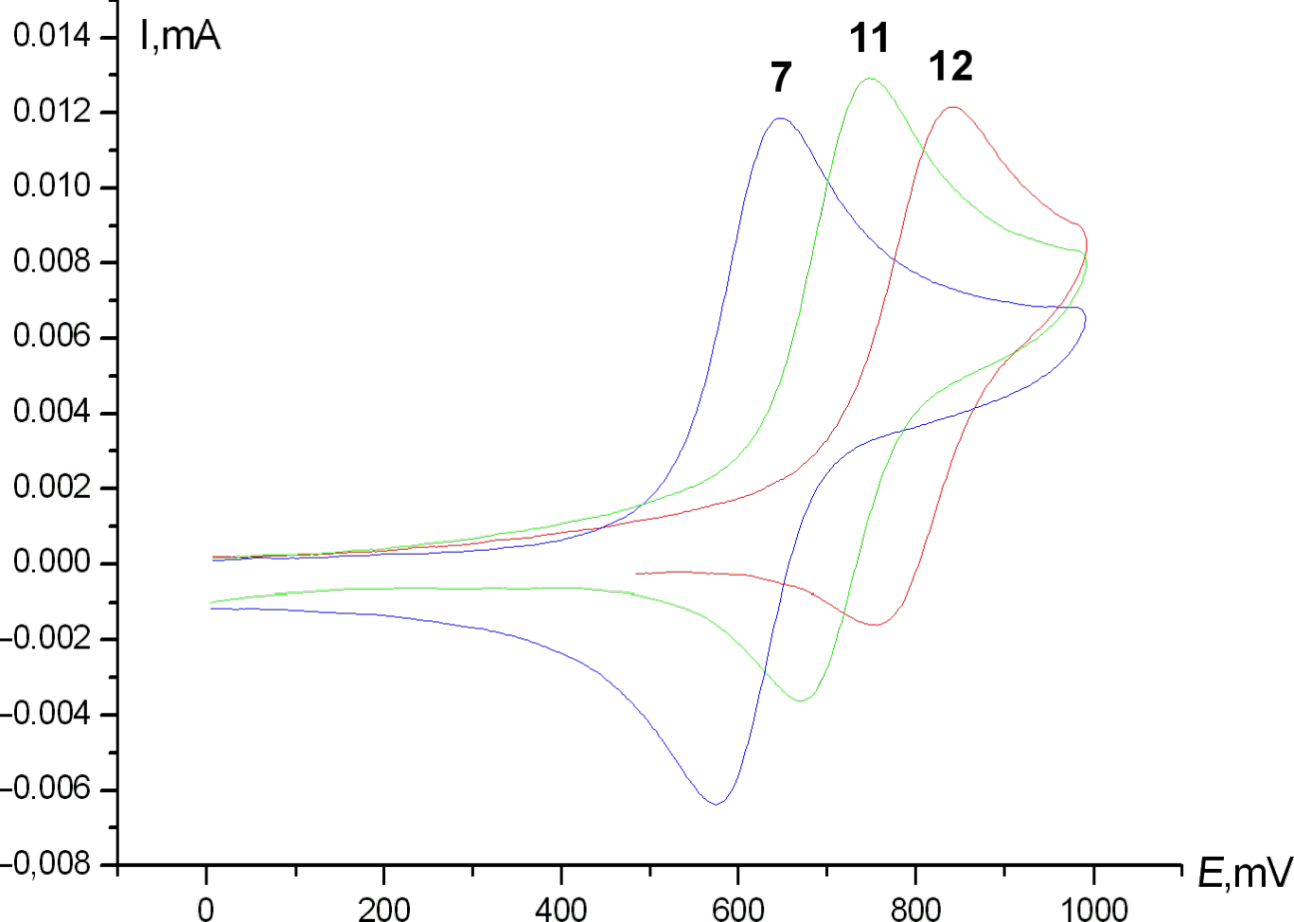
Typical voltammogramms of vinylferrocenes **7** (blue), **11** (green), **12** (red), anodic region.

All compounds are characterized by reversible oxidation waves in the range of 0.64–0.84 V that corresponds to the Fe^2+^/Fe^3+^ redox transformation. The values of the respective oxidation potential depend mainly on the amount of the vinyl groups attached to the ferrocene core. There is also the effect of the halogen atom, though to a lesser extent compared to the effect of the amount of vinyl groups. For example, all dichloro and dibromo compounds (**1**, **2**, **6**, and **7**, one vinyl fragment) are oxidized at potentials of 0.64–0.65 V, whereas for the corresponding tetrahalo compounds (**10** and **11**, two vinyl fragments) these values are shifted to more anodic potentials, namely, 0.74 V. The same is true for trifluoromethyl ferrocenes **4**, **8**, and **12** (Figure 1).

**Figure 2:**
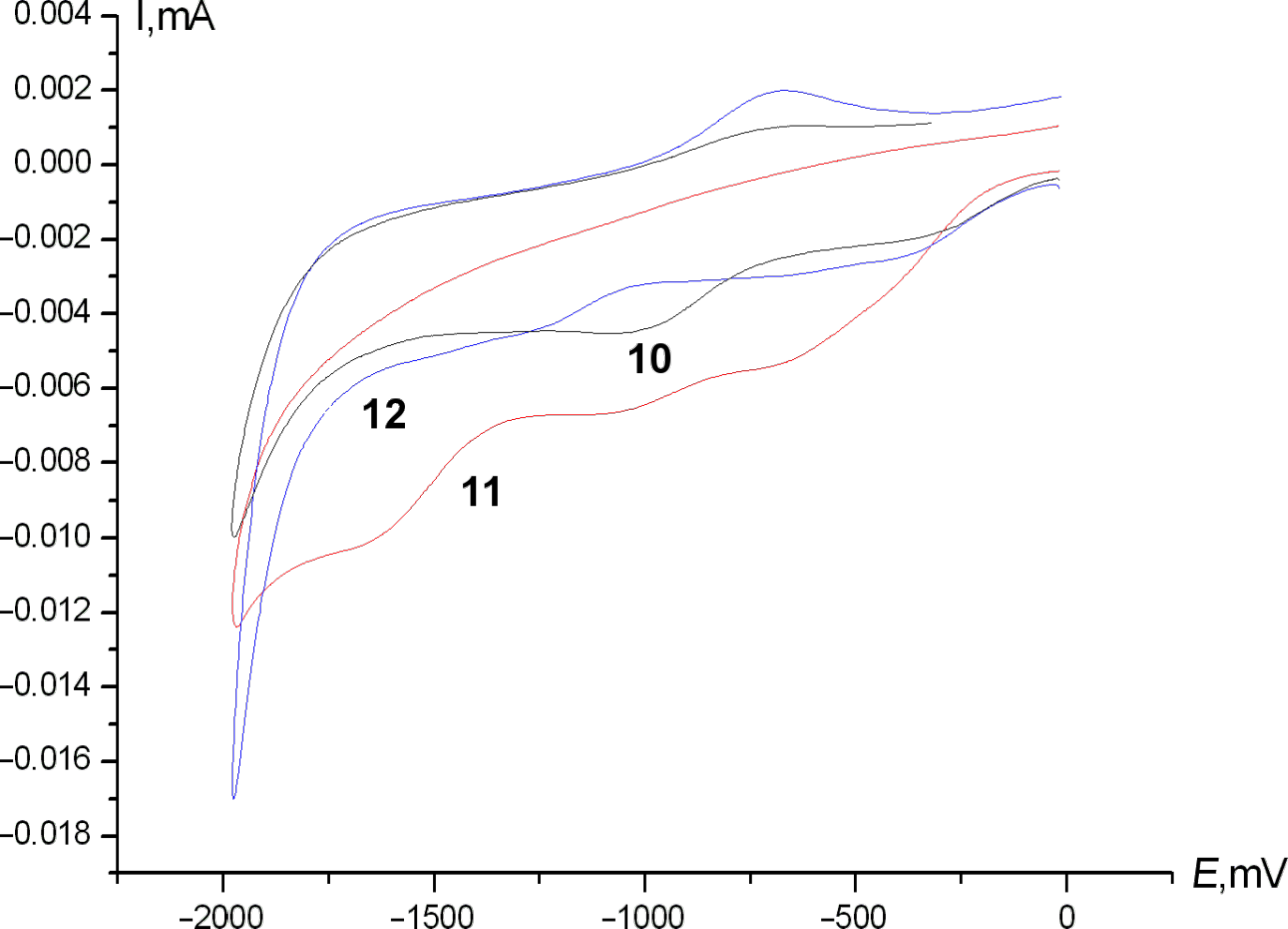
Typical voltammogramms of divinylferrocenes **10** (black), **11** (red), **12** (blue), cathodic region.

In the cathodic region, monovinylic compounds **1**, **2**, **4**, and **6**–**8** show no reduction up to 1.80 V, whilst the corresponding divinylic molecules **10**–**12** exhibit pronounced reduction waves in the range of 0.34–1.66 V (Figure 2).

The processes behind the cathodic waves are not totally clear at the moment, but we assume that the radical-ions formed after the first electron transfer would enter the intramolecular cyclization reaction involving the second adjacent double bond with subsequent electropolymerization. The latter is confirmed by a pronounced decrease in current values (3–4 times) as compared to current values of oxidation at the iron atom. These findings allow us to state that the synthesized molecules are promising starting materials for the electrochemical synthesis of ferrocene-containing conjugated polymers.

## Conclusion

In conclusion, a novel stereoselective route to ferrocenyl haloalkenes and bis-alkenes was elaborated on the basis of a catalytic olefination reaction of *N*-unsubstituted hydrazones obtained from ferrocene-containing aldehydes and ketones. Electrochemical properties of synthesized alkenes were investigated and promising electrochemical characteristics were demonstrated.

## Supporting Information

File 1 Experimental details, analytical data and copies of NMR spectra of all synthesized compounds, X-ray data of compound **8**.
