# Supplementary material for: A new approach to ferrocene derived alkenes via copper-catalyzed olefination
Source: Beilstein J Org Chem. 2015 Nov 3;11:2072–8. doi: 10.3762/bjoc.11.223 (PMC4660972; doi:10.3762/bjoc.11.223)
Supplement: File 1 — Experimental details, analytical data and copies of NMR spectra of all synthesized compounds, X-ray data of compound 8. [file Beilstein_J_Org_Chem-11-2072-s001.pdf]

Supporting Information

for

A new approach to ferrocene derived alkenes via copper-catalyzed olefination

Vasily M. Muzalevskiy[1], Aleksei V. Shastin[1,2], Alexandra D. Demidovich[1], Namiq G. Shikhaliev[3], Abel M. Magerramov[3], Victor N. Khrustalev[4,5], Rustem D. Rakhimov[1], Sergey Z. Vatsadze[1] and Valentine G. Nenajdenko[1,5]*

Address: [1]Department of Chemistry, Lomonosov Moscow State University, Moscow 119991, Russia, [2]Institute of Problems of Chemical Physics, Russian Academy of Sciences, Chernogolovka, Moscow region, 142432 Russia, [3]Baku State University, Department of Chemistry, Z. Xalilov Str. 23, Az 1148 Baku, Azerbaijan, [4]Peoples' Friendship University of Russia, 6 Miklukho-Maklay Street, Moscow 117198, Russia and [5]A.N. Nesmeyanov Institute of Organoelement Compounds, Russian Academy of Sciences, 28 Vavilov Street, Moscow 119991, Russia

Email: Valentine G. Nenajdenko* - nenajdenko@gmail.com

* Corresponding author

Experimental details, analytical data and copies of NMR spectra of all synthesized compounds, X-ray data of compound 8

Table of contents

General remarks S2–S3
Reaction of ferrocene carbaldehyde with polyhalogenalkanes S3
Reaction of acetylferrocene and 1,1'-diacetylferrocene hydrazones with polyhalogenalkanes S3
Compound characterization data S4–S9
Copies of all NMR spectra S10–S27
X-ray structure determination of **8** S28–S37

General remarks. All reactions were monitored by thin-layer chromatography carried out on Merck silica gel plates. Column chromatography was performed on silica gel (Merck, 63–200 mesh). ^{1}H NMR and ^{13}C NMR spectra were recorded on a Bruker AMX 400 spectrometer at 400 and 100 MHz, respectively. ^{19}F NMR spectra were recorded on an AGILENT 400-MR 400 spectrometer (377 MHz). Compounds were dissolved in $CDCl_3$, acetone-d_6 and benzene-d_6. Tetramethylsilane and C_6F_6 were used as internal standards. IR spectra were recorded on a ThermoNicolet IR 200 spectrometer. Mass spectra (HRMS (ESI)) were measured on a MicroTof Bruker Daltonics instrument. Electrochemical measurements were carried out using a IPC 2000 potentiostat. Platinum (d = 2.8 mm) discs pressed in Teflon served as working electrodes; a 0.05 M solution of Bu_4NBF_4 as the supporting electrolyte, and an Ag/AgCl/KCl(sat.) electrode was the reference electrode. All the measurements were carried out under argon.

Dimethylformamide for electrochemical measurements (reagent grade) was stirred with anhydrous K_2CO_3 (20 g L^{-1}) for 4 days at $<$20 °C, decanted from the solid phase, and then purified by successive refluxing and vacuum distillation (bp 42 °C, 10 Torr) over CaH_2 (10 g L^{-1}) and anhydrous $CuSO_4$ (10 g L^{-1}). The purified solvent was stored over 4 Å molecular sieves. All other reagents were of reagent grade and were either used as such or distilled prior to use. Ferrocene carbaldehyde [1], acetylferrocene [2], 1,1'-diacetylferrocene [3] and corresponding hydrazones [4] were prepared as it was previously published. The NMR data of **1** [5] and **2** [6] are in agreement with those in the literature.

Reaction of ferrocene carbaldehyde with polyhalogenalkanes (PHA). One neck 50 mL round bottomed flask was charged with 0.228 g (1 mmol) of ferrocene carbaldehyde, 10 mL of ethylene glycol, 0.25 mL (5 mmol) of $N_2H_4{\cdot}H_2O$ and stirred 1 h until the aldehyde disappeared (TLC control). Next, 0.38 mL (4.4 mmol) of 1,2-

ethylenediamine and 0.0086 g (0.05 mmol) of $CuCl_2 \cdot 2H_2O$ was added, stirred for 1–2 min and then 6 mmol of corresponding PHA was added in one portion under cooling with a crashed ice bath. The reaction mixture was maintained overnight at room temperature, poured into 50 mL of water and extracted with CH_2Cl_2 (3 × 20 mL). The combined extract was additionally washed with 20 mL of water and dried over Na_2SO_4. Solvents were evaporated in vacuo, the residue was purified by passing through a short silica gel pad using a 3:1 mixture of hexane and CH_2Cl_2 as an eluent.

Reaction of acetylferrocene and 1,1'-diacetylferrocene hydrazones with polyhalogenalkanes. 1 mmol of hydrazone, 10 mL of DMSO, 5 equiv of the corresponding base, and 0.1 equiv of CuCl were mixed together in a one neck 50 mL round bottomed flask and stirred for 5 min. Next, 5 mmol of the corresponding PHA was added in one portion under cooling with crashed ice bath. The reaction mixture was maintained overnight at room temperature and then quenched and purified by exactly the same steps as in case of ferrocene carbaldehyde olefination.

1-(2,2-Dichlorovinyl)ferrocene (1):

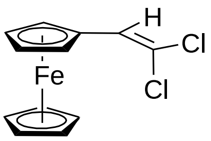

Red-brown crystalline compound; m.p. 52-54 °C; (174 mg, 62% yield); ^{1}H NMR (400.1 MHz, $CDCl_3$) δ 4.18 (s, 5H), 4.29 (t, J_{HH}= 1.9 Hz, 2H), 4.58 (t, J_{HH}= 1.9 Hz, 2H), 6.53 (s, 1H, $CH{=}CCl_2$); ^{13}C NMR ($CDCl_3$, 100.6 MHz) δ 69.1 (2 CH), 69.2 (7 CH), 77.7 (C_q), 116.1 ($CH{=}\underline{C}Cl_2$), 127.2 ($\underline{C}H{=}CCl_2$).

1-(2,2-Dibromovinyl)ferrocene (2):

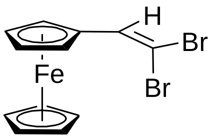

Red-brown crystalline compound; m.p. 62-63 °C; (139 mg, 38% yield); ^{1}H NMR (400.1 MHz, $CDCl_3$) δ 4.19 (s, 5H), 4.29 (t, J_{HH}= 1.9 Hz, 2H), 4.66 (t, J_{HH}= 1.9 Hz, 2H), 7.15 (s, 1H, $CH{=}CBr_2$); ^{13}C NMR ($CDCl_3$, 100.6 MHz) δ 69.0 (2 CH), 69.2 (2 CH), 69.3 (5 CH), 79.5 (C_q), 83.5 ($CH{=}\underline{C}Br_2$), 135.5 ($\underline{C}H{=}CBr_2$).

1-(2,3,3,3-Tetrafluoroprop-1-enyl)ferrocene (3):

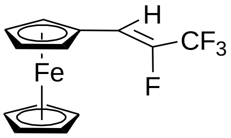

Red-brown oil, obtained as a mixture of *Z,E*-isomers (74:26); (137 mg, 46% yield); *Z*-isomer ^{1}H NMR (400.1 MHz, $CDCl_3$) δ 4.16 (s, 5H), 4.35 (s, 2H), 4.54 (s, 2H), 6.21 (d, $^{2}J_{HF}$ = 36.5 Hz, 1H); ^{13}C NMR ($CDCl_3$, 100.6 MHz) δ 69.5 (5 CH), 69.8 (d, $^{4}J_{CF}$= 3.7 Hz, 2 CH(cpd)), 70.1 (2 CH), 72.6 (C_q), 111.3 ($\underline{C}H{=}CF{-}CF_3$), 118.9 (q, $^{1}J_{CF}$= 271.3 Hz, CF_3); ^{19}F NMR (376.3 MHz, $CDCl_3$) δ -137.3-137.1 (m, 1F, F), -72.8 (d, $^{2}J_{FF}$=10.2 Hz, 3F, CF_3); *E*-isomer ^{1}H NMR (400.1 MHz, $CDCl_3$) δ 6.48 (d, $^{2}J_{HF}$ = 16.2 Hz, 1H); ^{13}C NMR ($CDCl_3$, 100.6 MHz) δ 69.4 (5 CH), 70.0 (2 CH), 70.6 (d, $^{4}J_{CF}$= 7.7 Hz, 2 CH(cpd)), 72.6 (C_q), 114.9 (dq, $^{2}J_{CF}$= 24.0 Hz, $^{3}J_{CF}$= 2.7 Hz, $\underline{C}H{=}CF{-}CF_3$); ^{19}F

NMR (376.3 MHz, $CDCl_3$) δ -129.7-129.9 (m, 1F, F), -67.9 (d, $^2J_{FF}$=5.5 Hz, 3F, CF_3); HRMS (ESI): m/z calcd for $C_{13}H_{10}F_4Fe$ [M+]: 298.0063; found: 298.0063.

1-(2-Bromo-3,3,3-trifluoroprop-1-enyl)ferrocene (4):

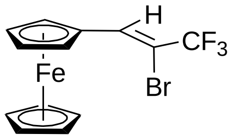

Red-brown oil, obtained as a mixture of *Z,E*-isomers (75:25), (152 mg, 42% yield); *Z*-isomer ^{1}H NMR (400.1 MHz, $CDCl_3$) δ 4.22 (s, 5H), 4.46 (s, 2H), 4.86 (s, 2H), 7.40 (s, 1H, $CH{=}CBrCF_3$); ^{13}C NMR ($CDCl_3$, 100.6 MHz) δ 69.61 (5 CH), 70.6 (2 CH), 70.7 (2 CH), 75.5 (C_q), 104.0 (q, $^2J_{CF}$= 36.9 Hz, C-CF_3), 121.4 (q, $^1J_{CF}$= 270.5 Hz, CF_3), 134.7 (q, $^3J_{CF}$= 4.8 Hz, CH=CBr-CF_3); ^{19}F NMR (376.3 MHz, $CDCl_3$) δ -67.1; *E*-isomer ^{1}H NMR (400.1 MHz, $CDCl_3$) δ 4.23 (s, 5H), 4.41 (s, 2H), 4.51 (s, 2H), 7.40 (s, 1H, $CH{=}CBrCF_3$); ^{13}C NMR ($CDCl_3$, 100.6 MHz) δ 69.57 (5 CH), 70.2 (q, $^5J_{CF}$= 3.0 Hz, 2 CH(cpd)), 70.8 (2 CH), 121.0 (q, $^1J_{CF}$= 272.0 Hz, CF_3), 140.4 (q, $^3J_{CF}$= 2.6 Hz, CH=CBr-CF_3); ^{19}F NMR (376.3 MHz, $CDCl_3$) δ -60.2; IR (cm^{-1}) v 1655 (C=C); HRMS (ESI): m/z calcd for $C_{13}H_{10}BrF_3Fe$ [M+]:357.9263; found: 357.9262.

1-(2-Chloro-3,3,3-trifluoroprop-1-enyl)ferrocene (5):

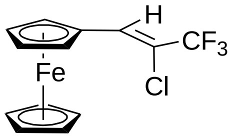

Red-brown oil, obtained as a mixture of *Z,E*-isomers (75:25), (131 mg, 42% yield); *Z*-isomer ^{1}H NMR (400.1 MHz, $CDCl_3$) δ 4.20 (s, 5H), 4.44 (s, 2H), 4.78 (s, 2H), 7.10 (s, 1H, $CH{=}CClCF_3$); ^{13}C NMR ($CDCl_3$, 100.6 MHz) δ 69.6 (5 CH), 70.65 (2 CH), 70.70 (2 CH), 74.6 (C_q), 114.7 (q, $^2J_{CF}$= 36.7 Hz, C-CF_3), 121.3 (q, $^1J_{CF}$= 270.9 Hz, CF_3), 131.2 (q, $^3J_{CF}$= 4.1 Hz, CH=CCl-CF_3); ^{19}F NMR (376.3 MHz, $CDCl_3$) δ -69.0; *E*-isomer ^{1}H NMR (400.1 MHz, $CDCl_3$) δ 4.40 (s, 2H), 4.49 (s, 2H), 6.92 (s, 1H, $CH{=}CClCF_3$); ^{13}C NMR ($CDCl_3$, 100.6 MHz) δ 70.2 (q, $^5J_{CF}$= 2.6 Hz, 2 CH(cpd)), 75.5

(C_q), 121.0 (q, $^{1}J_{CF}$= 272.0 Hz, CF_3), 136.1 (q, $^{3}J_{CF}$= 1.8 Hz, CH=CCl-CF_3); ^{19}F NMR (376.3 MHz, $CDCl_3$) δ -62.4; IR (cm^{-1}) v 1655 (C=O); HRMS (ESI): m/z calcd for $C_{13}H_{10}ClF_3Fe$ [M+]: 313.9767; found: 313.9774.

1-(1,1-Dichloroprop-1-en-2-yl)ferrocene (6):

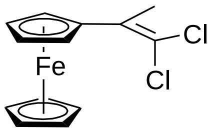

Red-brown crystalline compound; m.p. 48-49 °C; (105 mg, 35% yield); ^{1}H NMR (400.1 MHz, $CDCl_3$) δ 2.29 (s, 3H, CH_3), 4.21 (s, 5H), 4.33 (s, 2H), 4.62 (s, 2H); ^{13}C NMR ($CDCl_3$, 100.6 MHz) δ 21.8 (CH_3), 68.4 (2 CH), 68.9 (7 CH), 83.2 (C_q), 112.7 (C=CCl_2), 131.4 (C=CCl_2); HRMS (ESI): m/z calcd for $C_{13}H_{12}Cl_2Fe$ [M+]: 293.9661; found: 293.9668.

1-(1,1-Dibromoprop-1-en-2-yl)ferrocene (7):

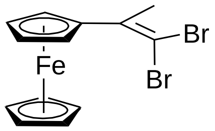

Red-brown crystalline compound; m.p. 59-61 °C; (190 mg, 50% yield); ^{1}H NMR (400.1 MHz, $CDCl_3$) δ 2.32 (s, 3H, CH_3), 4.20 (s, 5H), 4.30 (t, J_{Hh}= 1.9 Hz, 2H), 4.60 (t, J_{Hh}= 1.9 Hz, 2H); ^{13}C NMR ($CDCl_3$, 100.6 MHz) δ 26.1 (CH_3), 68.6 (2 CH), 69.4 (52 CH), 69.6 (2 CH), 83.1 (C_q), 85.7 (C=CBr_2), 138.3 (C=CBr_2); HRMS (ESI): m/z calcd for $C_{13}H_{12}Br_2Fe$ [M+]: 383.8631; found: 383.8629.

1-(3-Bromo-4,4,4-trifluorobut-2-en-2-yl)ferrocene (8):

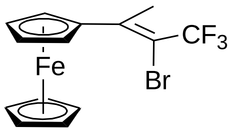

Red-brown crystalline compound; m.p. 55-57 °C; obtained as a mixture of *Z,E*-isomers (47:53), (235 mg, 64% yield); For the mixture of isomers ^{1}H NMR (400.1 MHz, $CDCl_3$) δ 2.41 (q, $^{5}J_{HF}$= 2.7 Hz, 3H, CH_3), 2.46 (q, $^{5}J_{HF}$= 1.9 Hz, 3H, CH_3), 4.20

(s, 5H), 4.21 (s, 5H), 4.34 (t, J_{HH}= 1.7 Hz, 2H), 4.39 (t, J_{HH}= 1.7 Hz, 2H), 4.41 (s, 2H), 4.69 (t, J_{HH}= 1.7 Hz, 2H); ^{13}C NMR ($CDCl_3$, 100.6 MHz) δ 22.0 (q, $^{4}J_{CF}$= 2.9 Hz, CH_3), 29.7 (CH_3), 69.1 (2 CH), 69.2 (4 CH), 69.5 (5 CH), 69.6 (5 CH), 70.3 (2 CH), 84.6 (C_q), 85.5 (C_q), 103.4 (q, $^{2}J_{CF}$= 36.1 Hz, $\underline{C}$-CF_3), 106.0 (q, $^{2}J_{CF}$= 37.6 Hz, $\underline{C}$-CF_3), 121.1 (q, $^{1}J_{CF}$= 272.2 Hz, CF_3), 121.8 (q, $^{1}J_{CF}$= 273.1 Hz, CF_3), 145.2 (q, $^{3}J_{CF}$= 2.2 Hz, $\underline{C}$Me=CBr-CF_3), 145.4 (q, $^{3}J_{CF}$= 2.6 Hz, $\underline{C}$Me=CBr-CF_3); ^{19}F NMR (376.3 MHz, $CDCl_3$) δ -56.4, -55.8; IR (cm^{-1}) v 1605 (C=C); HRMS (ESI): m/z calcd for $C_{14}H_{12}BrF_3Fe$ [M+]: 371.9420; found: 371.9412.

1-(3-Chloro-4,4,4-trifluorobut-2-en-2-yl)ferrocene (9):

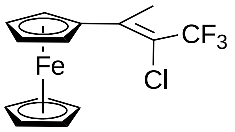

Red-brown oil; obtained as a mixture of *Z,E*-isomers (58:42); (75 mg, 22% yield); for the mixture of isomers ^{1}H NMR (400.1 MHz, acetone-d_6) δ 2.41 (q, $^{5}J_{HF}$= 2.8 Hz, 3H, CH_3), 2.47 (q, $^{5}J_{HF}$= 2.0 Hz, 3H, CH_3), 4.23 (s, 5H), 4.26 (s, 5H), 4.40 (t, J_{HH}= 1.8 Hz, 2H), 4.46 (t, J_{HH}= 1.8 Hz, 4H), 4.78 (t, J_{HH}= 1.9 Hz, 2H); ^{13}C NMR (acetone-d_6, 100.6 MHz) δ 20.6 (q, $^{4}J_{CF}$= 3.0 Hz, CH_3), 25.6 (CH_3), 70.1 (CH), 70.4 (CH), 70.5 (CH), 70.6 (CH), 71.2 (CH), 83.7 (C_q), 84.5 (C_q), 112.6 (q, $^{2}J_{CF}$= 36.5 Hz, $\underline{C}$-CF_3), 122.3 (q, $^{1}J_{CF}$= 271.6 Hz, CF_3), 122.9 (q, $^{1}J_{CF}$= 272.4 Hz, CF_3), 144.2 (q, $^{3}J_{CF}$= 1.7 Hz, $\underline{C}$Me=CCl-CF_3), 145.0 (q, $^{3}J_{CF}$= 2.0 Hz, $\underline{C}$Me=CCl-CF_3); ^{19}F NMR (376.3 MHz, acetone-d_6) δ -56.5, -56.7 (q, $^{5}J_{HF}$= 2.6 Hz); HRMS (ESI): m/z calcd for $C_{14}H_{12}ClF_3Fe$ [M+]: 327.9924; found: 327.9920.

1,1'-*bis*(1,1-Dichloroprop-1-en-2-yl)ferrocene (10):

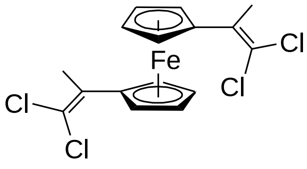

Red-brown viscous oil; (237 mg, 58% yield); ^{1}H NMR (400.1 MHz, $CDCl_3$) δ 2.26 (s, 6H, CH_3), 4.39 (s, 4H), 4.69 (s, 4H); ^{13}C NMR ($CDCl_3$, 100.6 MHz) δ 22.1 (2 CH_3), 71.1 (4 CH), 71.2 (4 CH), 84.9 (2 C_q), 113.4 (2 $C{=}\underline{C}Cl_2$), 132.7 (2 $\underline{C}{=}CCl_2$); IR (cm^{-1}) v 1589 (C=C); HRMS (ESI): m/z calcd for $C_{16}H_{14}Cl_4Fe$ [M+]: 403.9166; found: 403.9166.

1,1'-bis(1,1-Dibromoprop-1-en-2-yl)ferrocene (11):

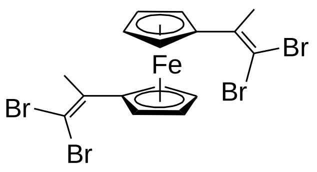

Red-brown crystalline compound; m.p. 59-61 °C; (427 mg, 74% yield); ^{1}H NMR (400.1 MHz, $CDCl_3$) δ 2.24 (s, 6H, CH_3), 4.35 (s, 4H), 4.68 (s, 4H); ^{13}C NMR ($CDCl_3$, 100.6 MHz) δ 25.9 (2 CH_3), 70.3 (4 CH), 70.9 (4 CH), 83.9 (2 C_q), 86.9 (2 $C{=}\underline{C}Br_2$), 137.4 (2 $\underline{C}{=}CBr_2$); IR (cm^{-1}) v 1562 (C=C); HRMS (ESI): m/z calcd for $C_{16}H_{14}Br_4Fe$ [M+]: 581.7134; found: 581.7136.

1,1'-bis(3-Bromo-4,4,4-trifluorobut-2-en-2-yl))ferrocene (12):

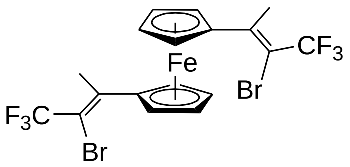

Red-brown liquid, obtained as a mixture of *Z,Z-Z,E-E,E* -isomers (~25:50:25), (365 mg, 65% yield); For the mixture of isomers ^{1}H NMR (400.1 MHz, C_6D_6) δ 2.10-2.19 (m, 6H, 2CH_3), 3.92-3.97 (m, 2H), 3.98-4.04 (m, 2H), 4.13-4.18 (m, 2H), 4.37-4.43 (m, 2H); ^{13}C NMR (C_6D_6, 100.6 MHz) δ 21.6 (CH_3), 21.7 (CH_3), 28.2 (CH_3), 28.3 (CH_3), 70.7 (CH), 70.8 (CH), 70.9 (5 CH), 71.0 (CH), 71.9 (CH), 85.8 (C_q), 85.9 (C_q), 86.58 (C_q), 86.63 (C_q), 104.5 (q, $^{2}J_{CF}$= 36.5 Hz, $\underline{C}$-CF_3), 104.6 (q, $^{2}J_{CF}$= 36.5 Hz, $\underline{C}$-CF_3), 106.9 (q, $^{2}J_{CF}$= 37.2 Hz, $\underline{C}$-CF_3), 107.1 (q, $^{2}J_{CF}$= 37.2 Hz, $\underline{C}$-CF_3), 121.8 (q, $^{1}J_{CF}$= 272.4 Hz, CF_3), 122.37 (q, $^{1}J_{CF}$= 273.1 Hz, CF_3), 122.41 (q, $^{1}J_{CF}$= 273.1 Hz, CF_3), 144.8 (q, $^{3}J_{CF}$= 1.5 Hz, $\underline{C}$Me=CBr-CF_3), 144.9 (q, $^{3}J_{CF}$= 1.8 Hz, $\underline{C}$Me=CBr-CF_3),

145.1 (<u>C</u>Me=CBr-CF_3); ^{19}F NMR (376.3 MHz, C_6D_6) δ -55.7 (q, $^{5}J_{HF}$= 2.7 Hz), -55.6 (q, $^{5}J_{HF}$= 2.7 Hz,) -54.9; IR (cm^{-1}) v 1597 (C=C); HRMS (ESI): m/z calcd for $C_{18}H_{14}Br_2F_6Fe$ [M+]: 559.8692; found: 559.8692.

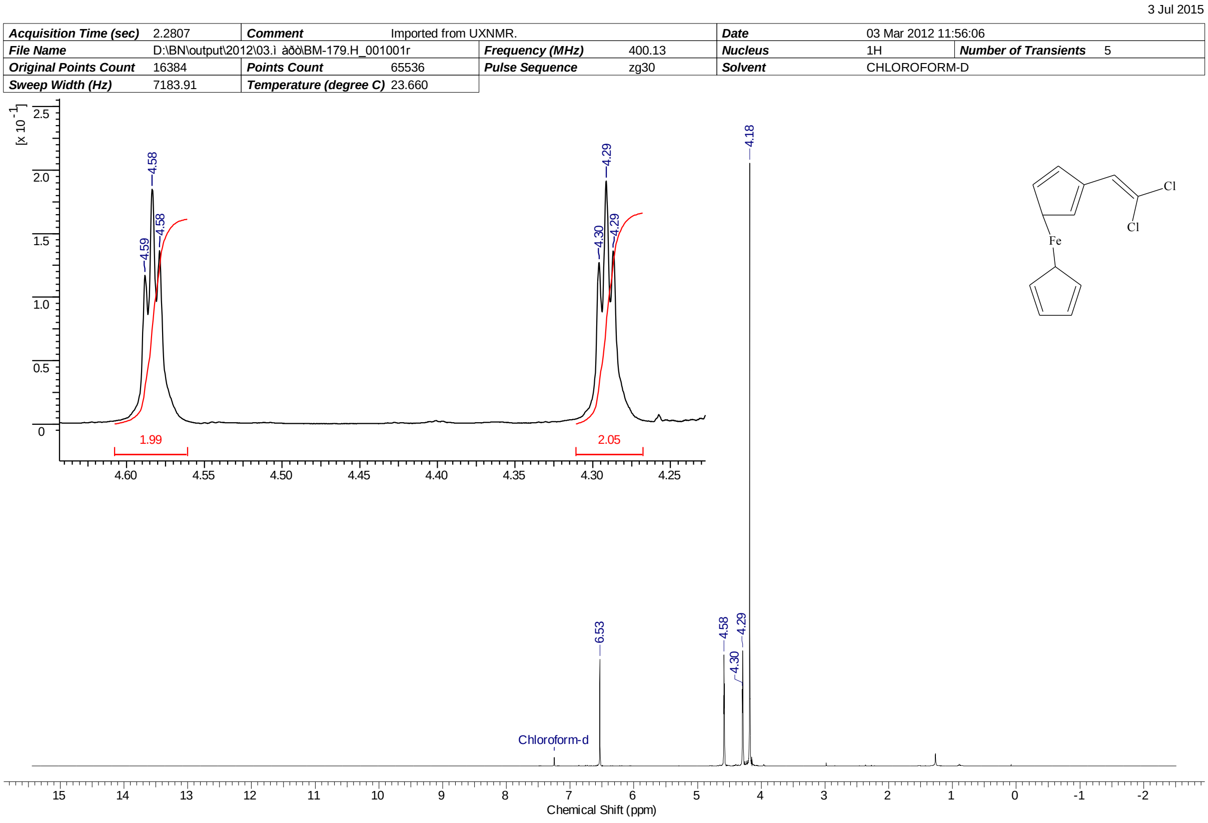

^{1}H NMR spectrum of **1** (400.1 MHz, $CDCl_3$)

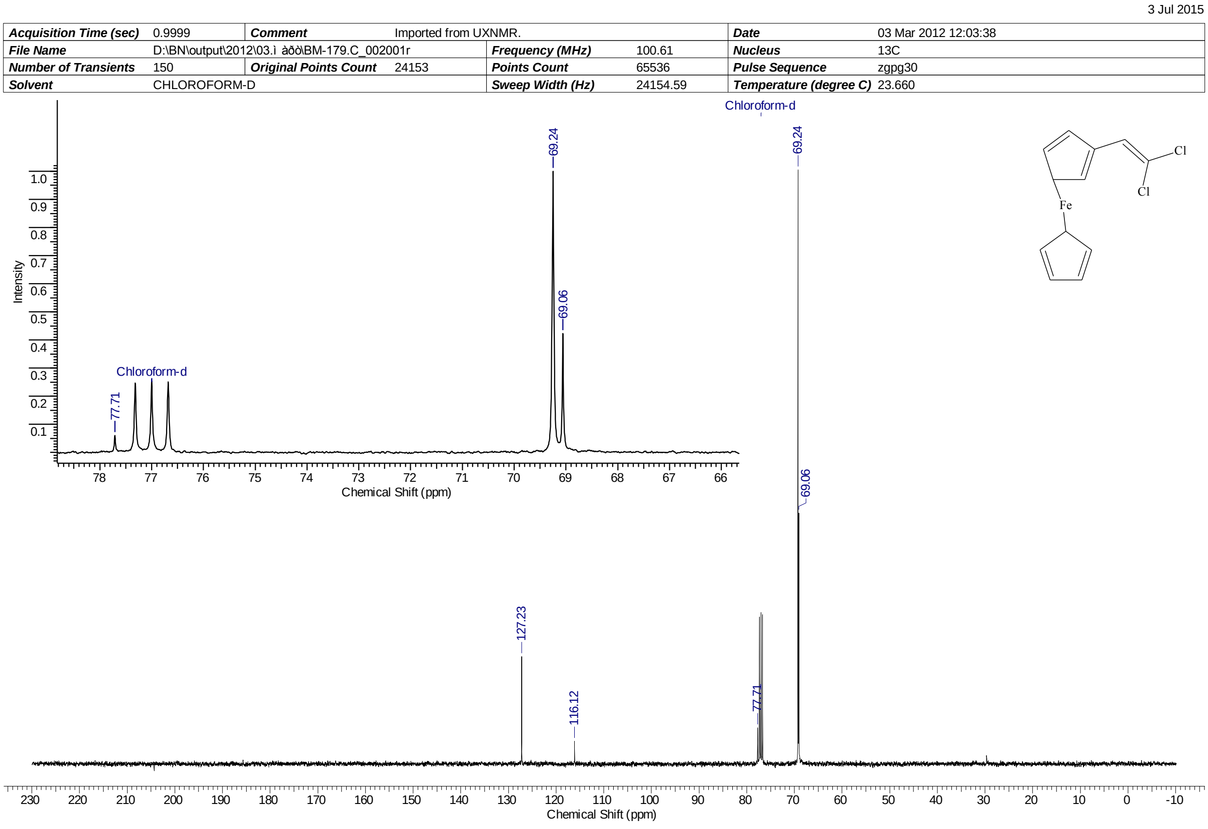

^{13}C NMR spectrum of **1** (100.6 MHz, $CDCl_3$)

16 May 2014

Acquisition Time (sec)	2.5559	Comment	Imported from UXNMR.			Date	15 May 2014 19:12:18		
File Name	D:\BN\output\2014\05.i àé\BM-485.H_001001r			Frequency (MHz)	400.13	Nucleus	1H	Number of Transients	4
Original Points Count	16384	Points Count	65536	Pulse Sequence	zg30	Solvent	CHLOROFORM-D		
Sweep Width (Hz)	6410.26	Temperature (degree C)	27.000						

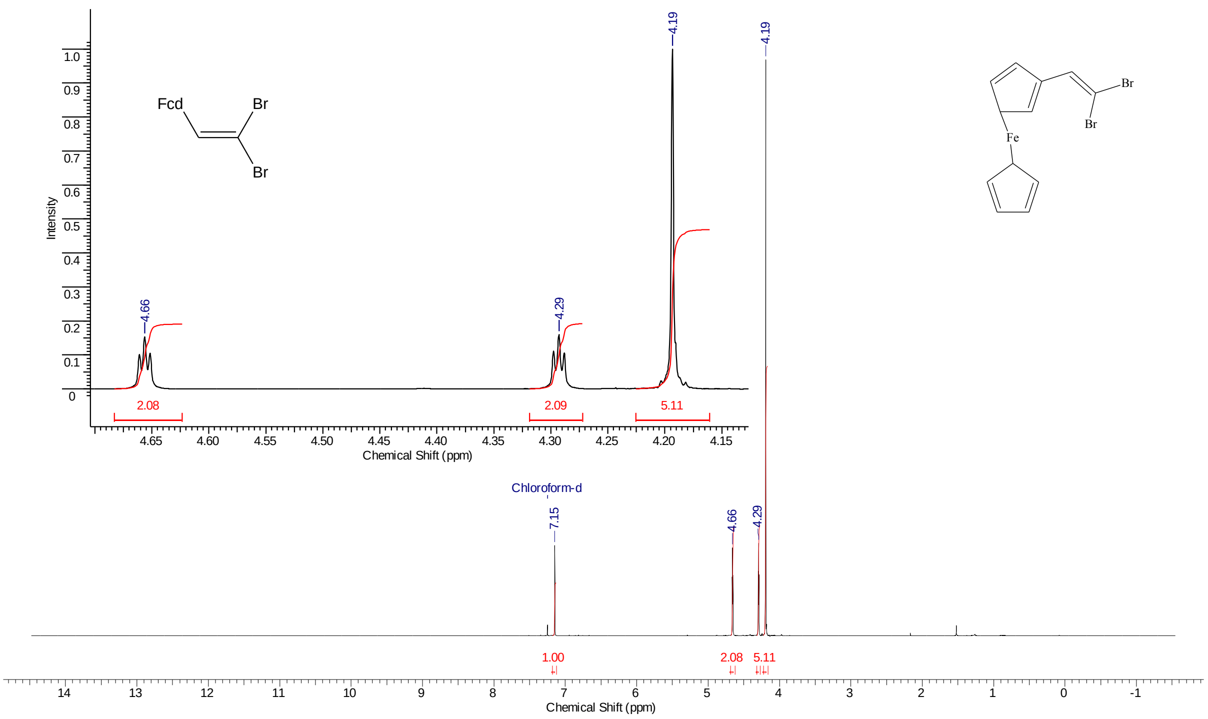

^{1}H NMR spectrum of **2** (400.1 MHz, $CDCl_3$)

16 May 2014

Acquisition Time (sec)	0.4999	Comment	Imported from UXNMR.			Date	15 May 2014 19:15:48
File Name	D:\BN\output\2014\05.i àé\BM-485.C_002001r			Frequency (MHz)	100.61	Nucleus	13C
Number of Transients	65	Original Points Count	12076	Points Count	65536	Pulse Sequence	zgpg30
Solvent	DEUTERIUM OXIDE			Sweep Width (Hz)	24154.59	Temperature (degree C)	27.000

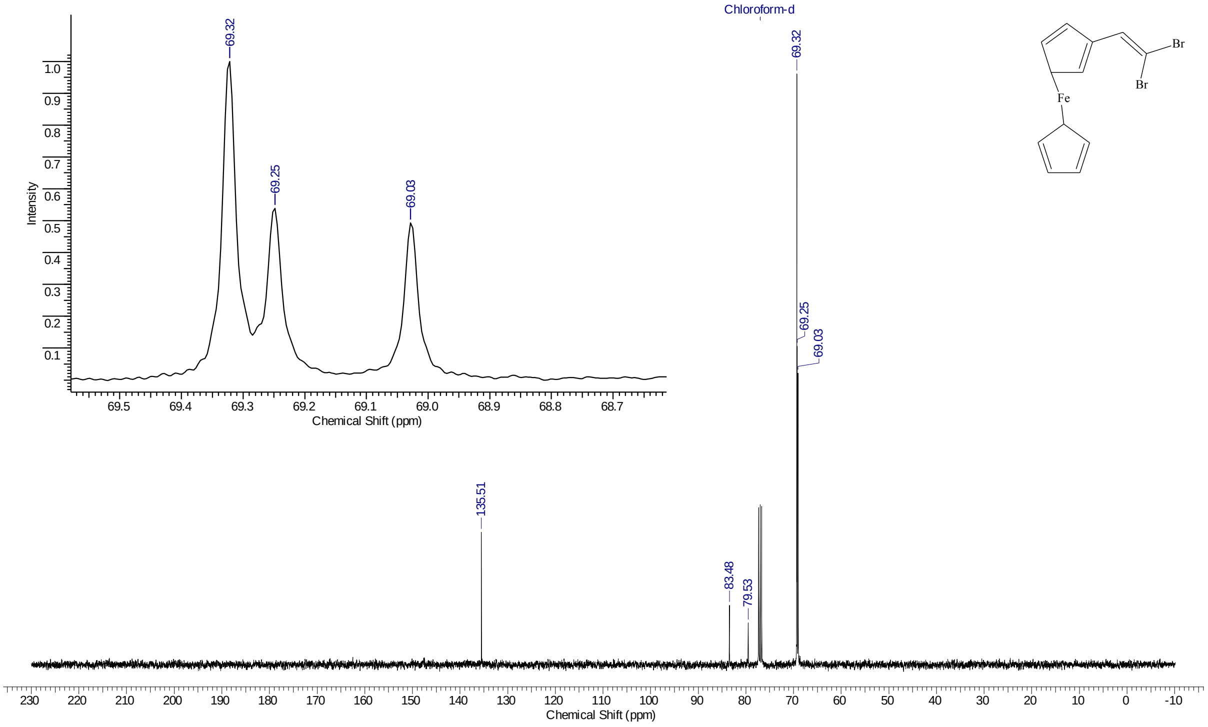

^{13}C NMR spectrum of **2** (100.6 MHz, $CDCl_3$)

2 Jul 2015

Acquisition Time (sec)	2.5559	***Comment***	Imported from UXNMR.	***Date***	09 Dec 2014 15:25:54
File Name	D:\BN\Docs (BN)\vasiliy\Manusr\Belstein_Ferrocene\SPEC_Fcd\SD-016.H_001001r			***Frequency (MHz)***	400.13
Nucleus	1H	***Number of Transients***	5 ***Original Points Count*** 16384	***Points Count***	65536
Pulse Sequence	zg30	***Solvent***	CHLOROFORM-D	***Sweep Width (Hz)***	6410.26
Temperature (degree C)	27.000				

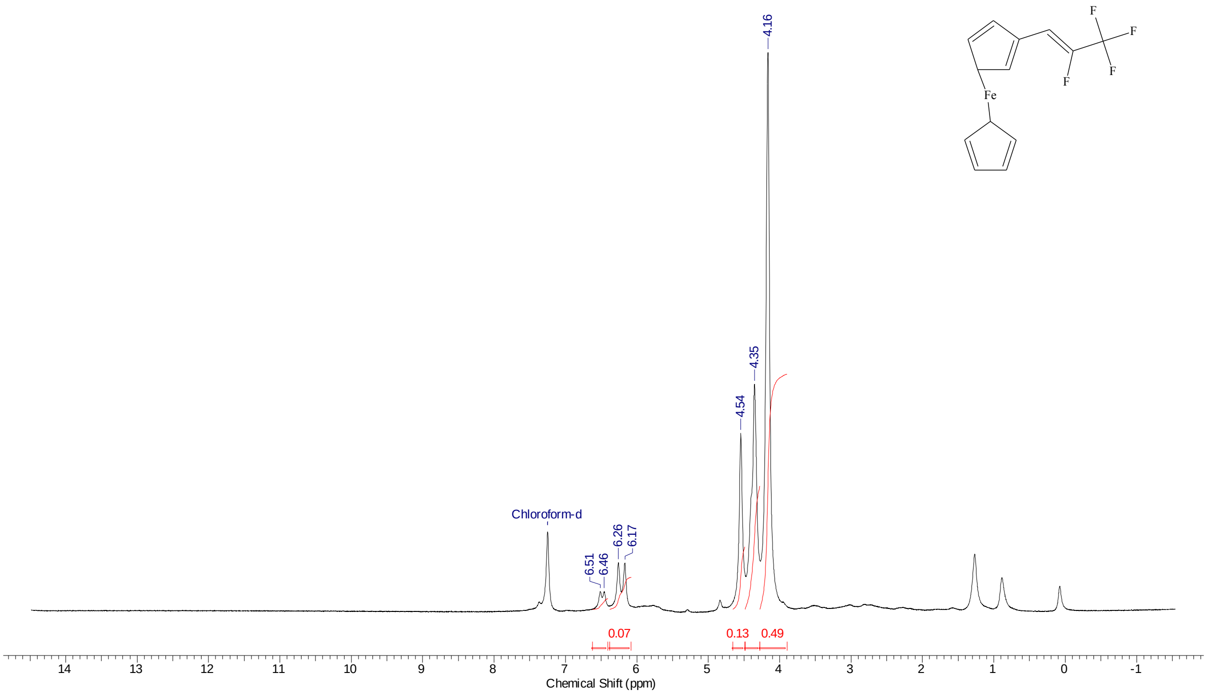

^{1}H NMR spectrum of **3** (400.1 MHz, $CDCl_3$)

2 Jul 2015

Acquisition Time (sec)	0.4999	***Comment***	Imported from UXNMR.	***Date***	09 Dec 2014 15:50:08
File Name	D:\BN\Docs (BN)\vasiliy\Manusr\Belstein_Ferrocene\SPEC_Fcd\SD-016.C_002001r			***Frequency (MHz)***	100.61
Nucleus	13C	***Number of Transients***	848 ***Original Points Count*** 12076	***Points Count***	65536
Pulse Sequence	zgpg30	***Solvent***	CHLOROFORM-D	***Sweep Width (Hz)***	24154.59
Temperature (degree C)	27.000				

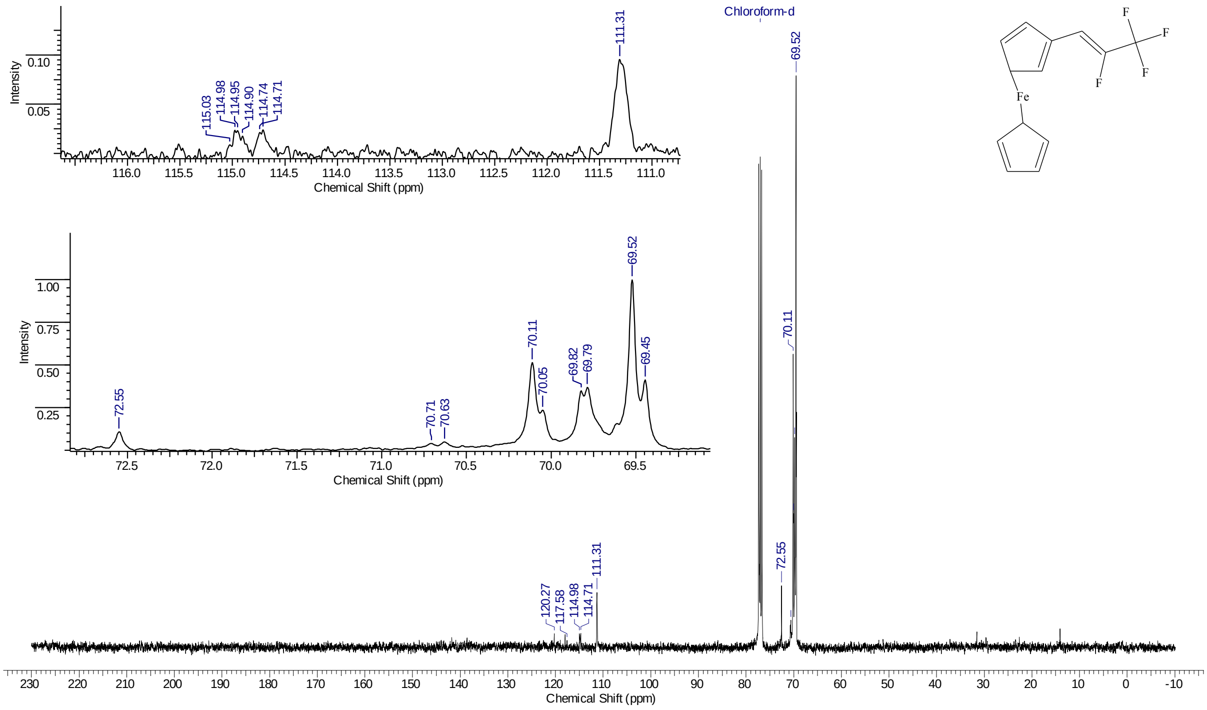

^{13}C NMR spectrum of **3** (100.6 MHz, $CDCl_3$)

2 Jul 2015

Acquisition Time (sec)	1.0000	***Date***	Dec 9 2014				
File Name	D:\BN\Docs (BN)\vasiliy\Manusr\Belstein_Ferrocene\SPEC_Fcd\19F\SD-016_20141209_01\FLUORINE_01					***Frequency (MHz)***	376.31
Nucleus	19F	***Number of Transients***	16	***Original Points Count***	89286	***Points Count***	131072
Pulse Sequence	s2pul	***Solvent***	CHLOROFORM-D	***Sweep Width (Hz)***	89285.71	***Temperature (degree C)***	40.000

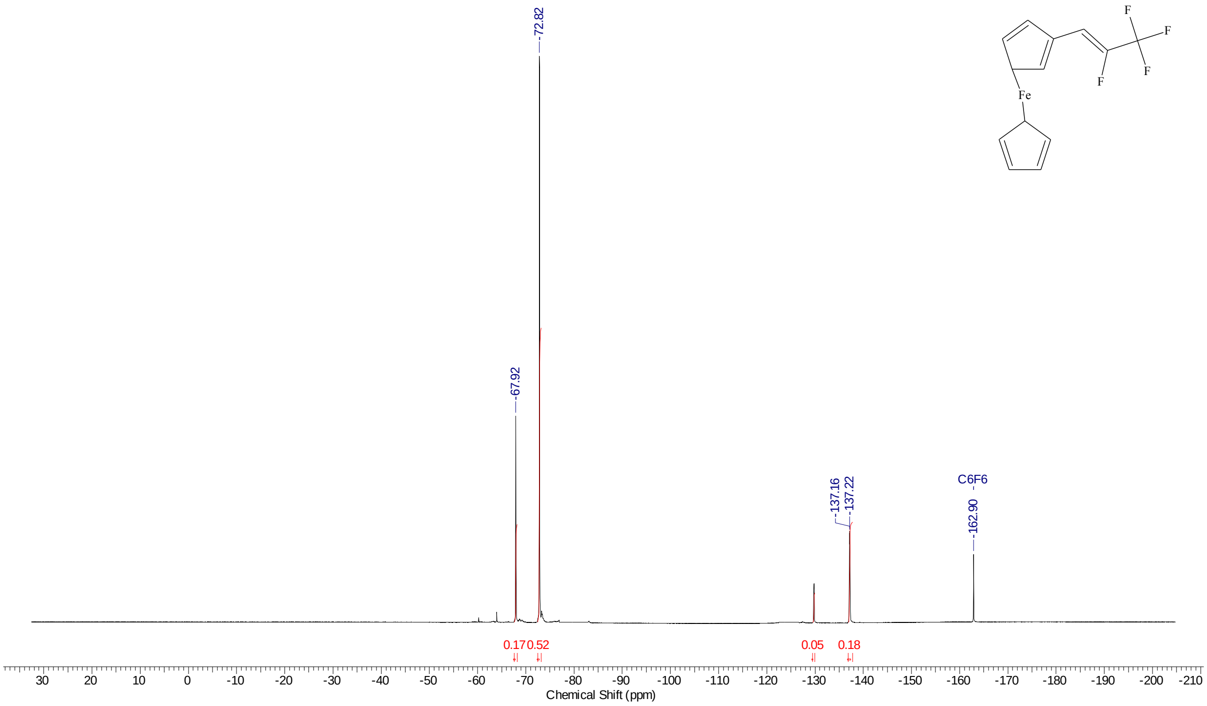

^{19}F NMR spectrum of **3** (376.3 MHz, $CDCl_3$)

2 Jul 2015

FW	358.9627	Formula	$C_{13}H_{10}BrF_3Fe$

Acquisition Time (sec)	2.5559	Comment	Imported from UXNMR.			Date	13 Nov 2014 20:09:44
File Name	D:\BN\Docs (BN)\vasiliy\Manusr\Belstein_Ferrocene\SPEC_Fcd\SD-012.H_001001r					Frequency (MHz)	400.13
Nucleus	1H	Number of Transients	4	Original Points Count	16384	Points Count	65536
Pulse Sequence	zg30	Solvent	DMSO-D6	Sweep Width (Hz)	6410.26	Temperature (degree C)	27.000

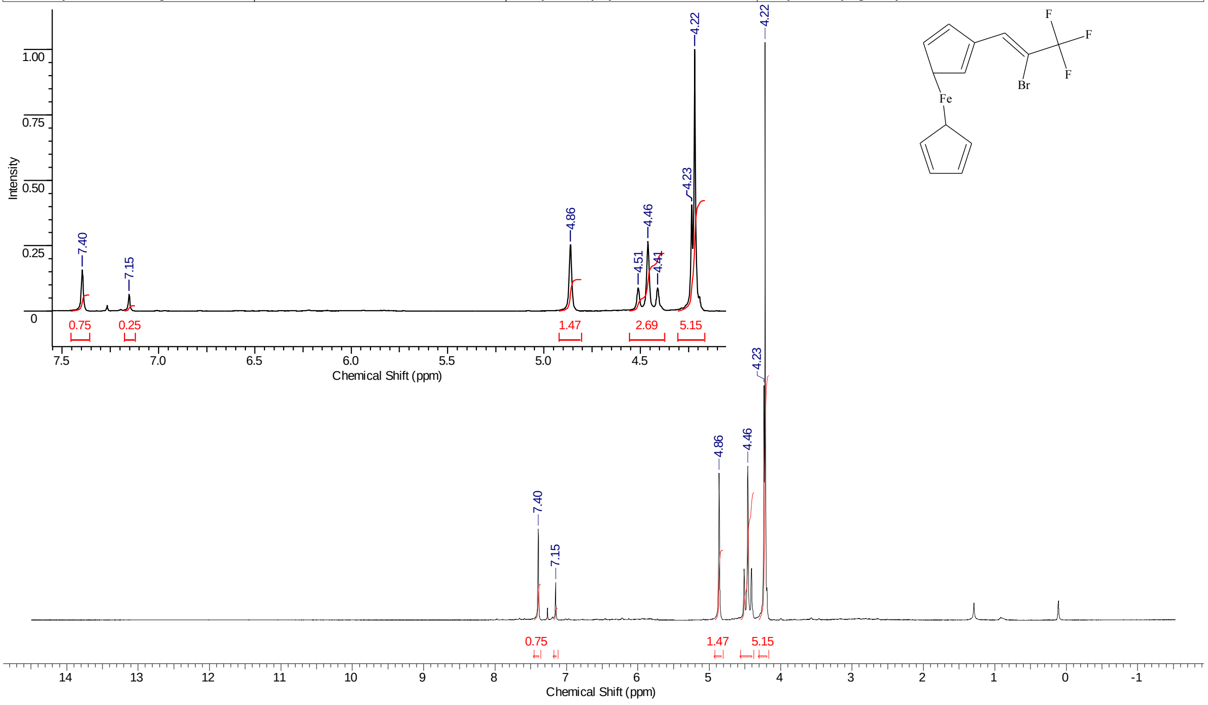

^{1}H NMR spectrum of **4** (400.1 MHz, $CDCl_3$)

2 Jul 2015

FW	358.9627	Formula	$C_{13}H_{10}BrF_3Fe$

Acquisition Time (sec)	0.4999	Comment	Imported from UXNMR.			Date	13 Nov 2014 20:13:52
File Name	D:\BN\Docs (BN)\vasiliy\Manusr\Belstein_Ferrocene\SPEC_Fcd\SD-012.C_002001r					Frequency (MHz)	100.61
Nucleus	13C	Number of Transients	131	Original Points Count	12076	Points Count	65536
Pulse Sequence	zgpg30	Solvent	DEUTERIUM OXIDE			Sweep Width (Hz)	24154.59
Temperature (degree C)	27.000						

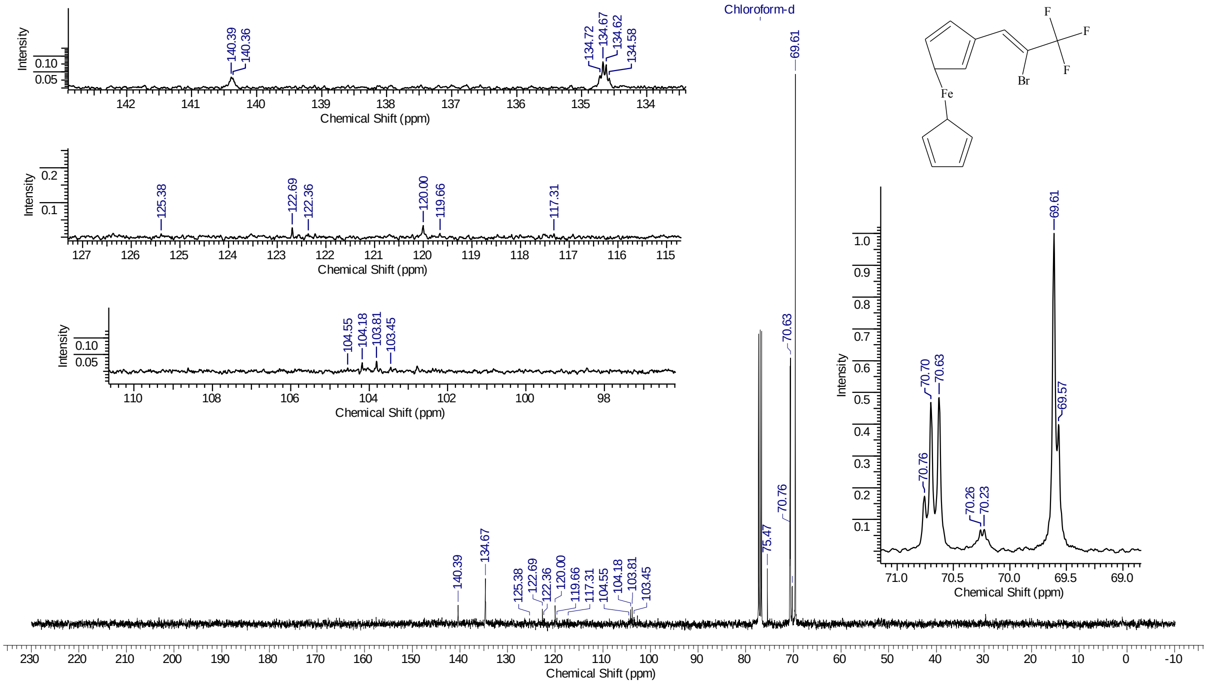

^{13}C NMR spectrum of **4** (100.6 MHz, $CDCl_3$)

FW	358.9627	Formula	$C_{13}H_{10}BrF_3Fe$

Acquisition Time (sec)	1.0000	Date	Nov 14 2014				
File Name	D:\BN\Docs (BN)\vasiliy\Manusr\Belstein_Ferrocene\SPEC_Fcd\19F\SD-012_20141114_01\FLUORINE_01					Frequency (MHz)	376.31
Nucleus	19F	Number of Transients	16	Original Points Count	89286	Points Count	131072
Pulse Sequence	s2pul	Solvent	CHLOROFORM-D	Sweep Width (Hz)	89285.71	Temperature (degree C)	50.000

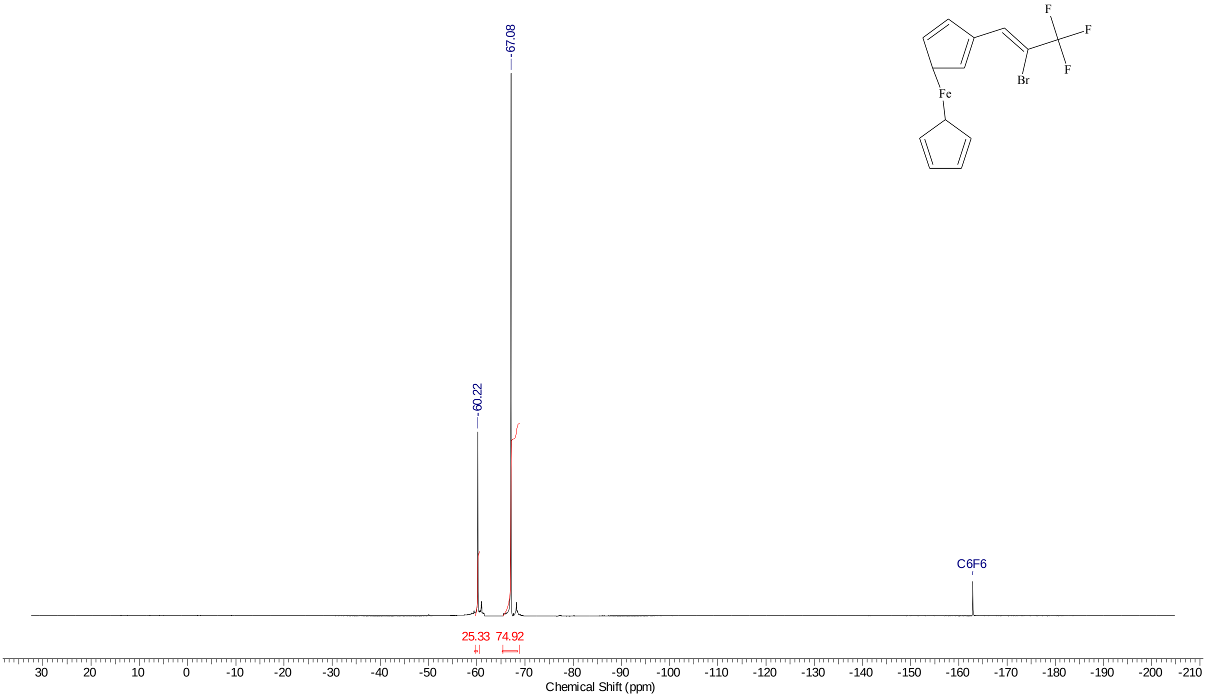

^{19}F NMR spectrum of **4** (376.3 MHz, $CDCl_3$)

2 Jul 2015

FW	314.5114	Formula	$C_{13}H_{10}ClF_3Fe$

Acquisition Time (sec)	2.5559	Comment	Imported from UXNMR.	Date	18 Nov 2014 15:18:30
File Name	D:\BN\Docs (BN)\vasiliy\Manusr\Belstein_Ferrocene\SPEC_Fcd\SD-013.H_001001r			Frequency (MHz)	400.13
Nucleus	1H	Number of Transients	12 / Original Points Count 16384	Points Count	65536
Pulse Sequence	zg30	Solvent	CHLOROFORM-D	Sweep Width (Hz)	6410.26
Temperature (degree C)	27.000				

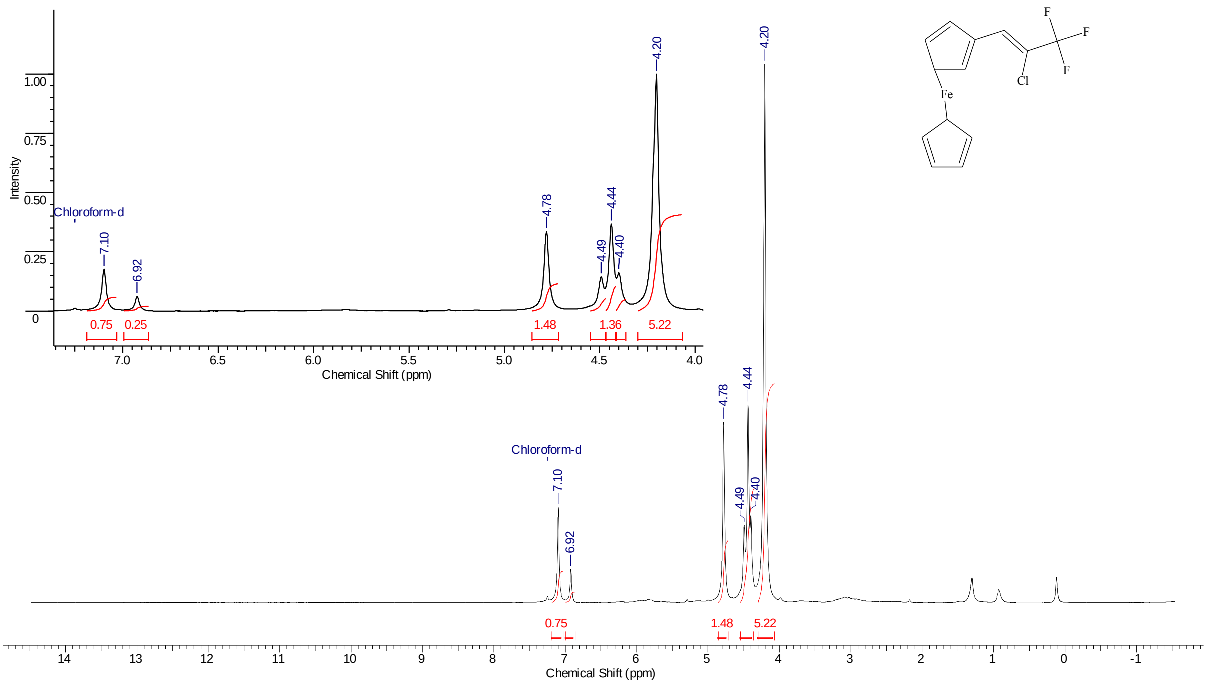

^{1}H NMR spectrum of **5** (400.1 MHz, $CDCl_3$)

2 Jul 2015

FW	314.5114	Formula	$C_{13}H_{10}ClF_3Fe$

Acquisition Time (sec)	0.4999	Comment	Imported from UXNMR.	Date	18 Nov 2014 15:25:16
File Name	D:\BN\Docs (BN)\vasiliy\Manusr\Belstein_Ferrocene\SPEC_Fcd\SD-013.C_002001r			Frequency (MHz)	100.61
Nucleus	13C	Number of Transients	234 / Original Points Count 12076	Points Count	65536
Pulse Sequence	zgpg30	Solvent	CHLOROFORM-D	Sweep Width (Hz)	24154.59
Temperature (degree C)	27.000				

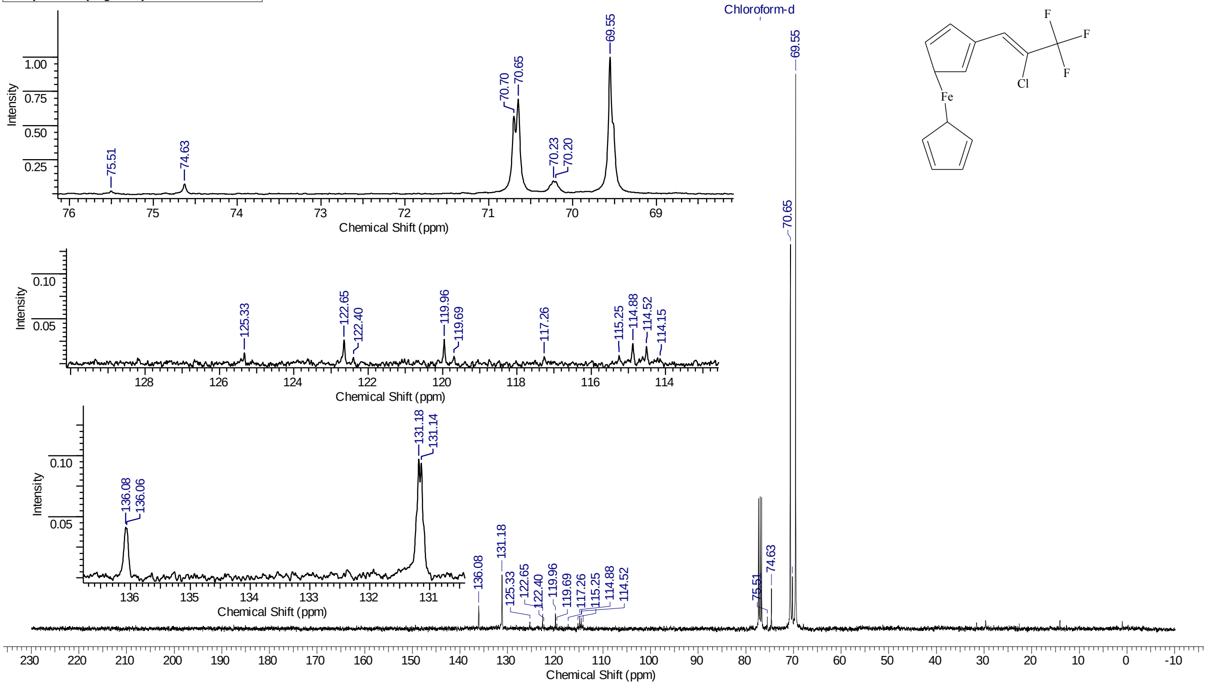

^{13}C NMR spectrum of **5** (100.6 MHz, $CDCl_3$)

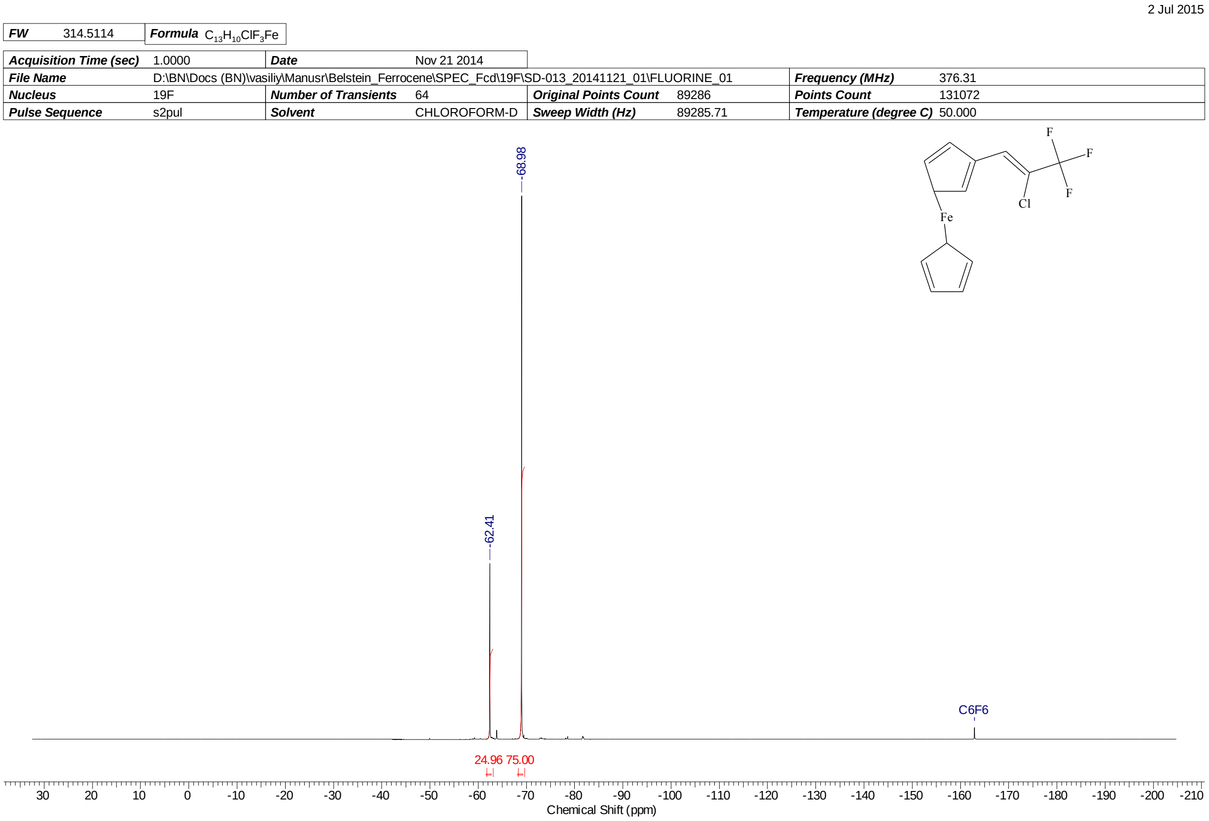

^{19}F NMR spectrum of **5** (376.3 MHz, $CDCl_3$)

Acquisition Time (sec)	2.2807	Comment	Imported from UXNMR.	Date	10 Mar 2012 13:46:22		
File Name	D:\NMR\OUTPUT\2012\03.ì àðò\BM-181.H_001001r	Frequency (MHz)	400.13	Nucleus	1H	Number of Transients	4
Original Points Count	16384	Points Count	65536	Pulse Sequence	zg30	Solvent	CHLOROFORM-D
Sweep Width (Hz)	7183.91	Temperature (degree C)	24.960				

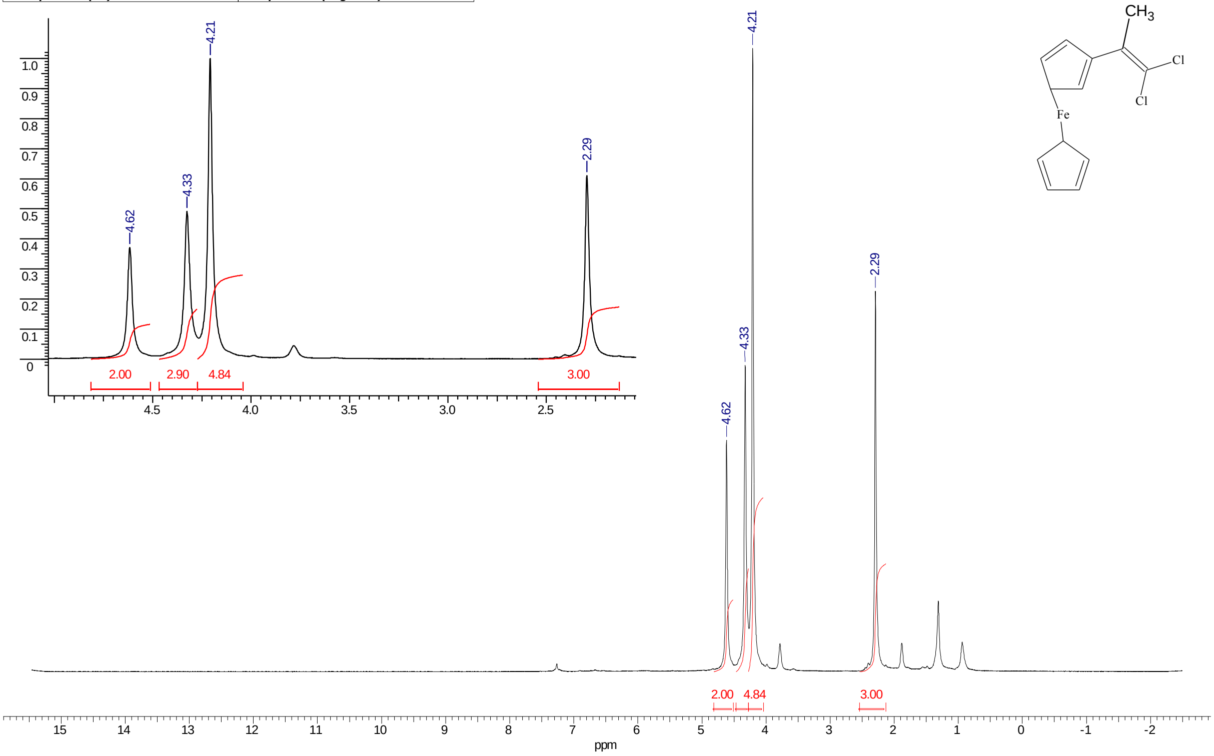

^{1}H NMR spectrum of **6** (400.1 MHz, $CDCl_3$)

Acquisition Time (sec)	0.9999	Comment	Imported from UXNMR.	Date	10 Mar 2012 13:31:34		
File Name	D:\NMR\OUTPUT\2012\03.ì àðò\BM-181.C_002001r	Frequency (MHz)	100.61	Nucleus	13C	Number of Transients	101
Original Points Count	24153	Points Count	65536	Pulse Sequence	zgpg30	Solvent	CHLOROFORM-D
Sweep Width (Hz)	24154.59	Temperature (degree C)	24.960				

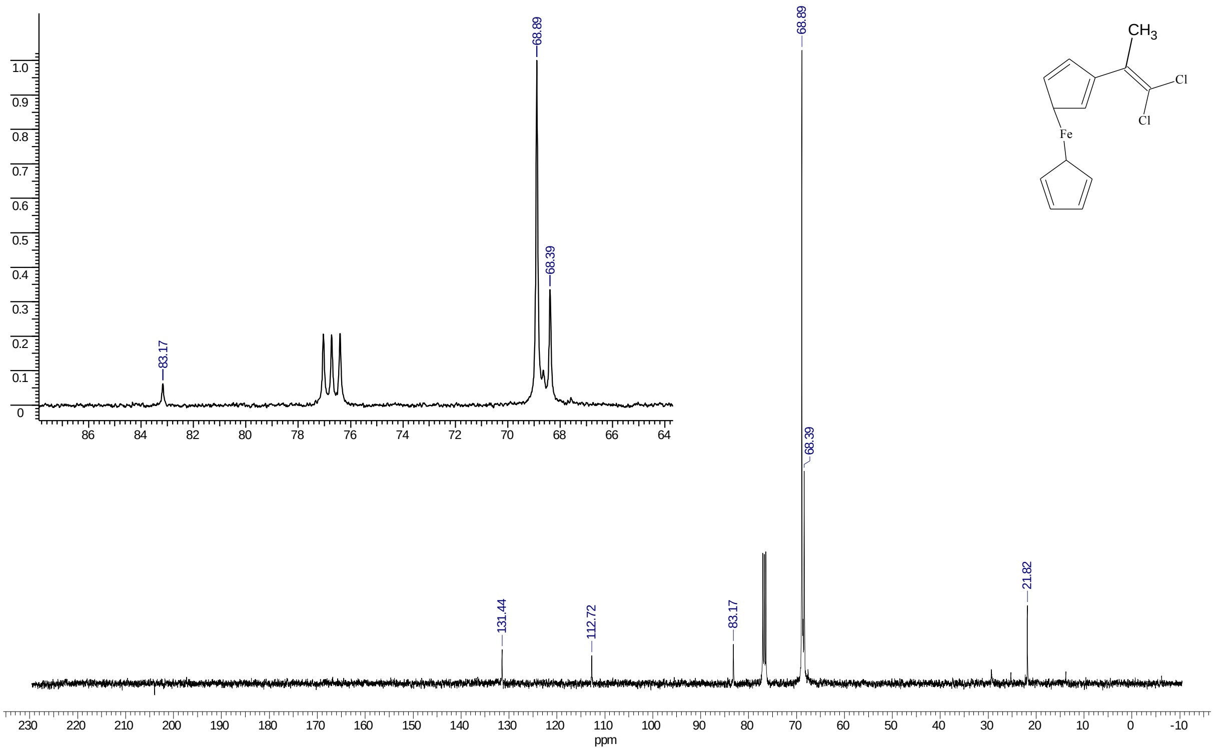

^{13}C NMR spectrum of **6** (100.6 MHz, $CDCl_3$)

Acquisition Time (sec)	2.2807	Comment	Imported from UXNMR.			Date	19 Apr 2012 21:29:28		
File Name	D:\NMR\OUTPUT\2012\04.àï ðàëü\SN4.H_001001r			Frequency (MHz)	400.13	Nucleus	1H	Number of Transients	3
Original Points Count	16384	Points Count	65536	Pulse Sequence	zg30	Solvent	CHLOROFORM-D		
Sweep Width (Hz)	7183.91	Temperature (degree C)	23.760						

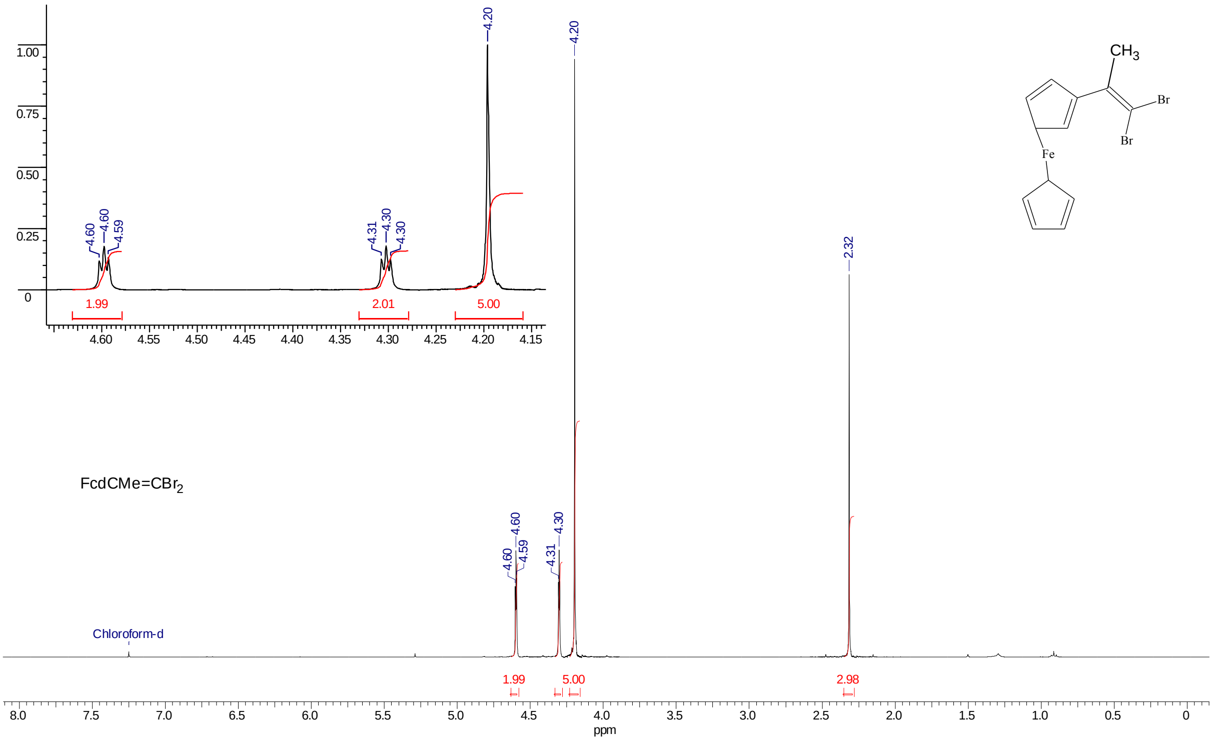

^{1}H NMR spectrum of **7** (400.1 MHz, $CDCl_3$)

Acquisition Time (sec)	0.9999	Comment	Imported from UXNMR.			Date	24 Apr 2012 16:48:04		
File Name	D:\NMR\OUTPUT\2012\04.àï ðàëü\SN4.C_002001r			Frequency (MHz)	100.61	Nucleus	13C	Number of Transients	97
Original Points Count	24153	Points Count	65536	Pulse Sequence	zgpg30	Solvent	CHLOROFORM-D		
Sweep Width (Hz)	24154.59	Temperature (degree C)	25.360						

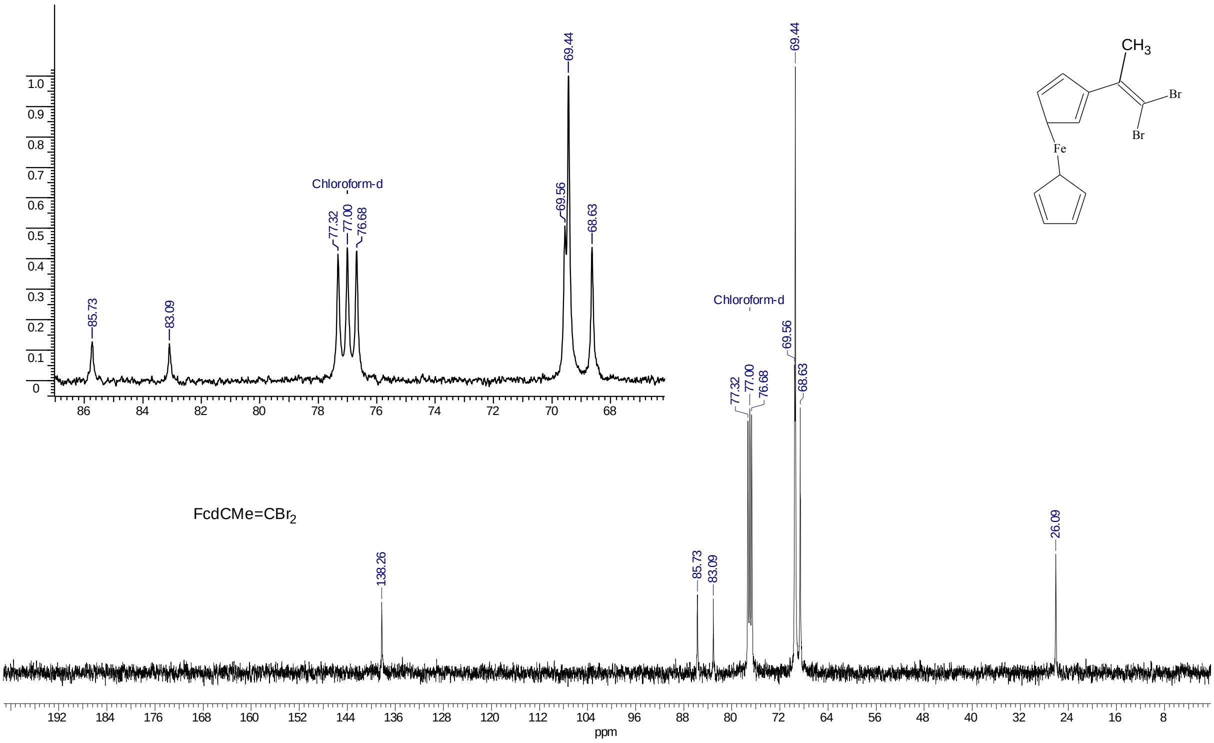

^{13}C NMR spectrum of **7** (100.6 MHz, $CDCl_3$)

4 Jul 2015

Acquisition Time (sec)	2.2807	***Comment***	Imported from UXNMR.			***Date***	24 Apr 2012 15:33:12
File Name	D:\BN\Docs (BN)\vasiliy\Manusr\Belstein_Ferrocene\SPEC_Fcd\SN8.H_001001r					***Frequency (MHz)***	400.13
Nucleus	1H	***Number of Transients***	9	***Original Points Count***	16384	***Points Count***	65536
Pulse Sequence	zg30	***Solvent***	CHLOROFORM-D			***Sweep Width (Hz)***	7183.91
Temperature (degree C)	25.260						

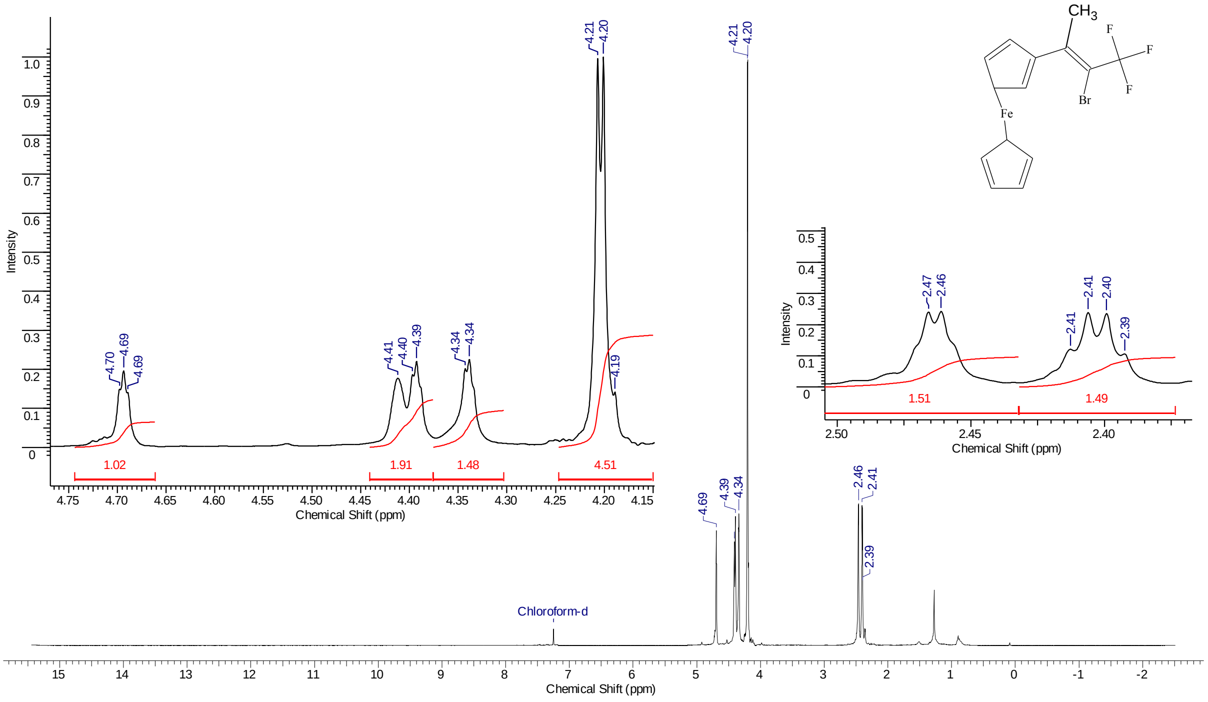

^{1}H NMR spectrum of **8** (400.1 MHz, $CDCl_3$)

4 Jul 2015

Acquisition Time (sec)	0.9999	***Comment***	Imported from UXNMR.			***Date***	25 Apr 2012 12:34:08
File Name	D:\BN\Docs (BN)\vasiliy\Manusr\Belstein_Ferrocene\SPEC_Fcd\SN8.C_002001r					***Frequency (MHz)***	100.61
Nucleus	13C	***Number of Transients***	200	***Original Points Count***	24153	***Points Count***	65536
Pulse Sequence	zgpg30	***Solvent***	CHLOROFORM-D			***Sweep Width (Hz)***	24154.59
Temperature (degree C)	24.260						

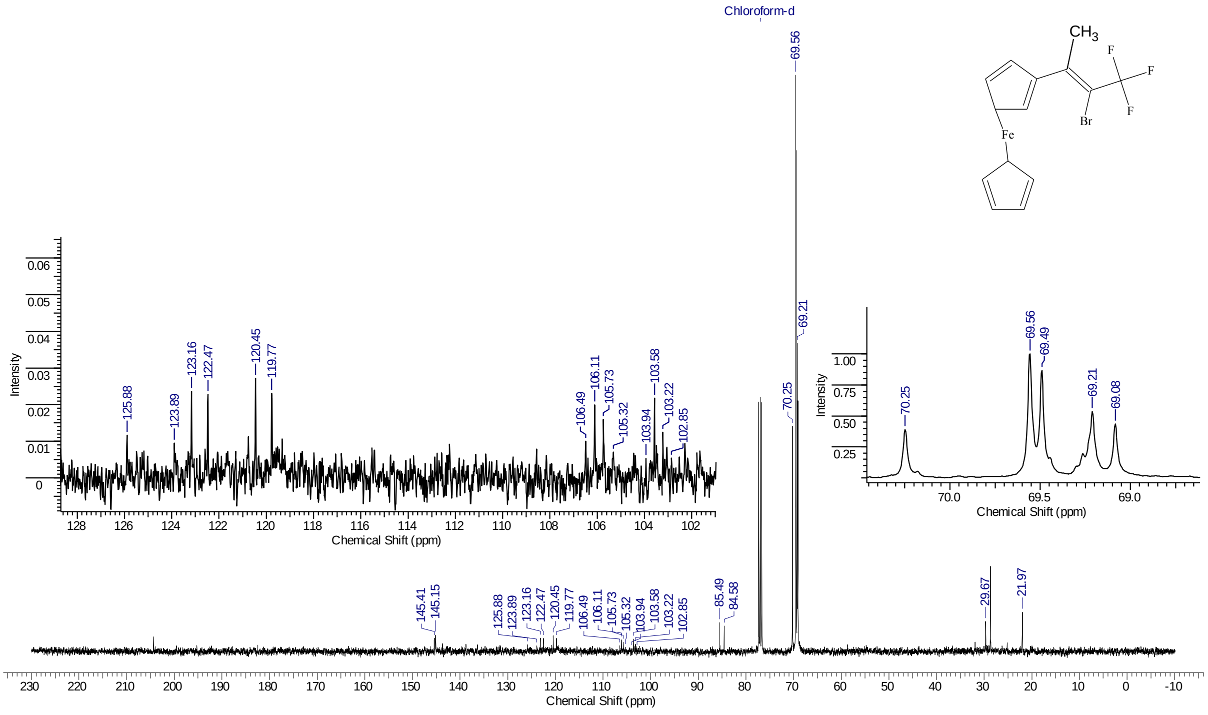

^{13}C NMR spectrum of **8** (100.6 MHz, $CDCl_3$)

4 Jul 2015

Acquisition Time (sec)	0.2449	***Comment***	/volc L26881.019 CDCl3; 300.0K; 17.04.2012			***Date***	25 Apr 2012 11:50:24
File Name	D:\BN\Docs (BN)\vasiliy\Manusr\Belstein_Ferrocene\SPEC_Fcd\19F\SN-8.{19F}\50549024.{19F}\50549024.{19F}_019000fid					***Frequency (MHz)***	282.39
Nucleus	19F	***Number of Transients***	1	***Original Points Count***	34018	***Points Count***	65536
Pulse Sequence	zg	***Solvent***	CHLOROFORM-D	***Sweep Width (Hz)***	138888.89	***Temperature (degree C)***	27.500

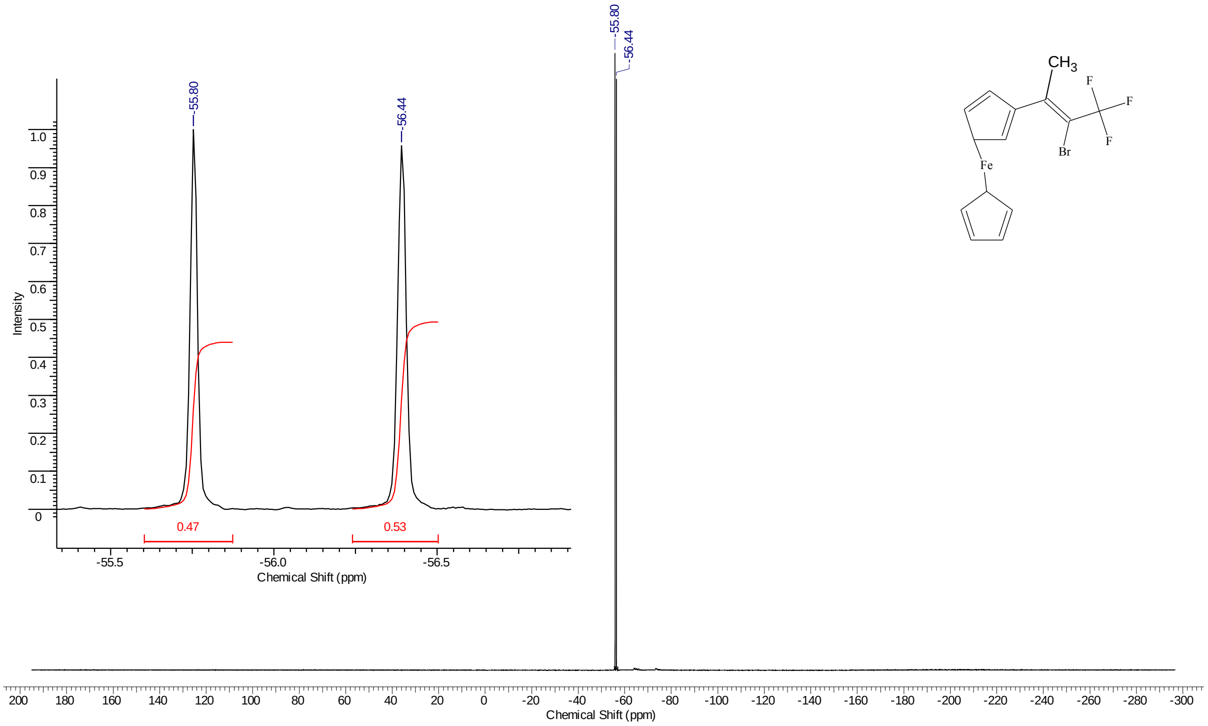

^{19}F NMR spectrum of **8** (376.3 MHz, $CDCl_3$)

10 Jul 2015

Acquisition Time (sec)	5.0000	***Date***	Jul 9 2015	***File Name***	D:\BN\output\F19\2015.07.09\BM-SO-029_20150709_01\PROTON_01				
Frequency (MHz)	399.97	***Nucleus***	1H	***Number of Transients***	8	***Original Points Count***	30048	***Points Count***	32768
Pulse Sequence	s2pul	***Solvent***	acetone	***Sweep Width (Hz)***	6009.62	***Temperature (degree C)***	26.000		

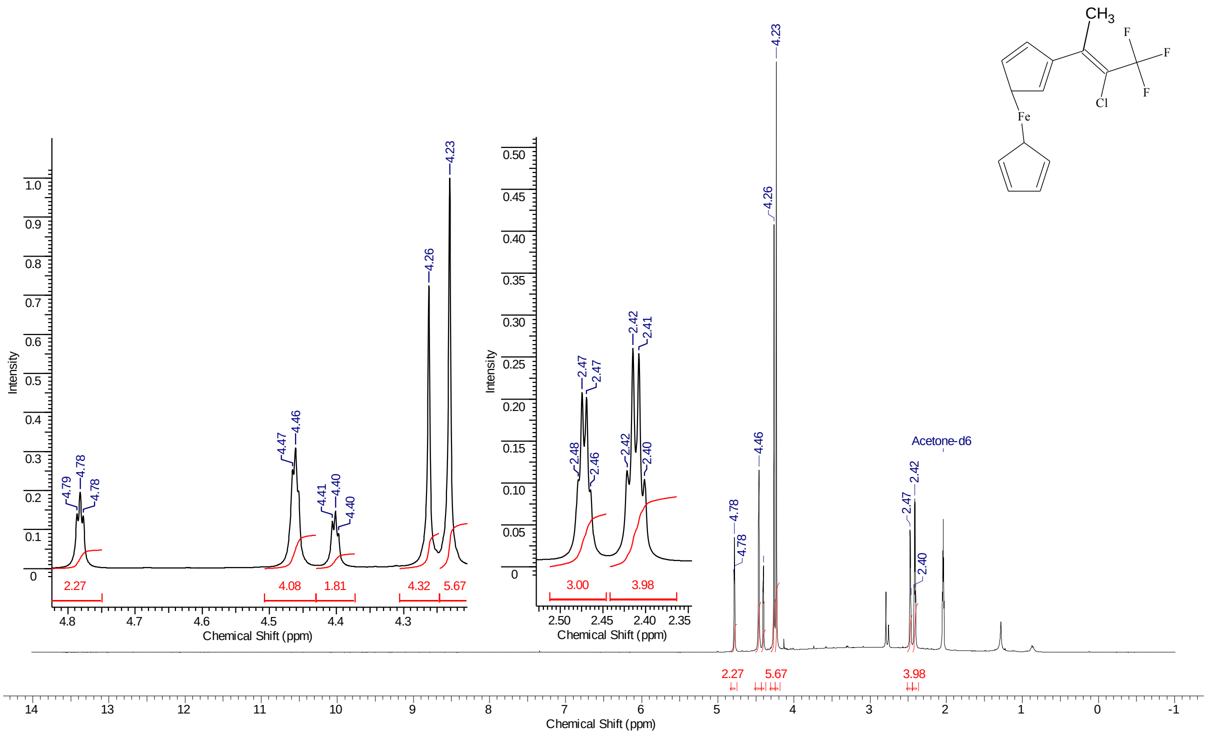

^{1}H NMR spectrum of **9** (400.1 MHz, acetone-d_6)

10 Jul 2015

Acquisition Time (sec)	1.5000	***Date***	Jul 9 2015	***File Name***	D:\BN\output\F19\2015.07.09\BM-SO-029-C_20150709_01\CARBON_01				
Frequency (MHz)	100.58	***Nucleus***	13C	***Number of Transients***	600	***Original Points Count***	40761	***Points Count***	65536
Pulse Sequence	s2pul	***Solvent***	acetone	***Sweep Width (Hz)***	27173.91	***Temperature (degree C)***	26.000		

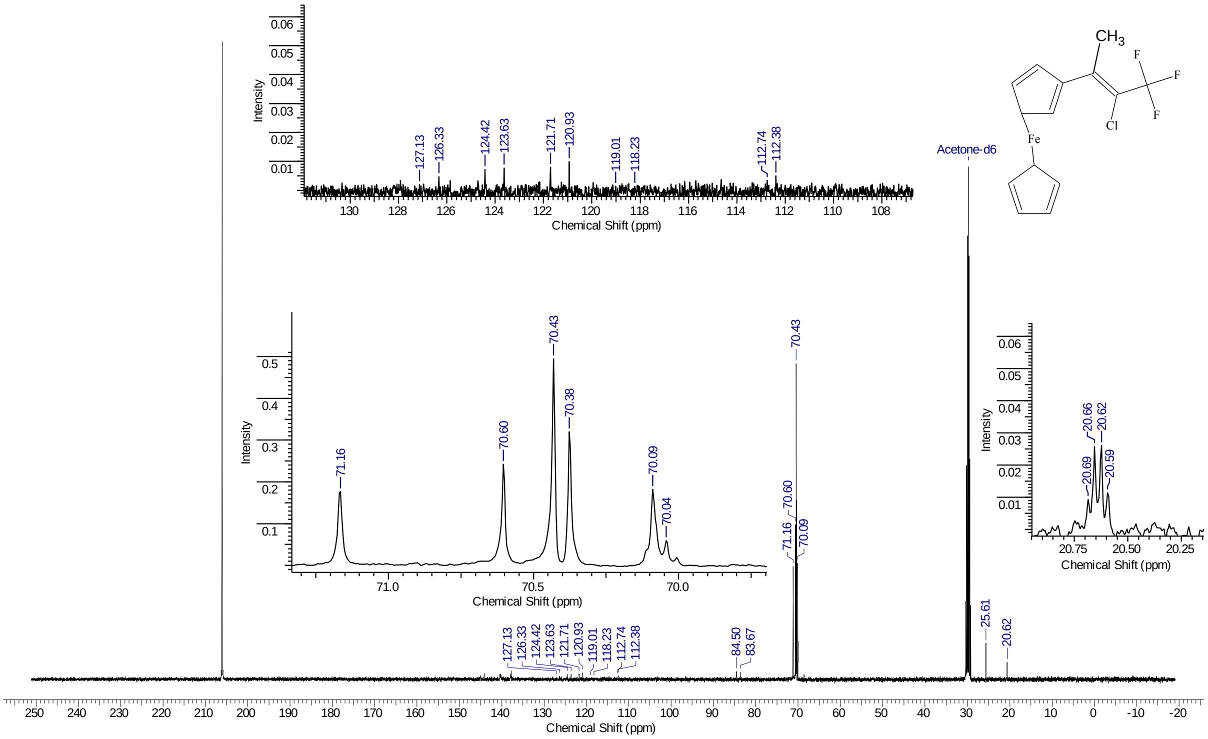

^{13}C NMR spectrum of **9** (100.6 MHz, acetone-d_6)

10 Jul 2015

Acquisition Time (sec)	3.3554	Date	Jul 9 2015	File Name	D:\BN\output\F19\2015.07.09\BM-SO-029-F_20150709_01\FLUORINE_01				
Frequency (MHz)	376.31	Nucleus	19F	Number of Transients	8	Original Points Count	262144	Points Count	262144
Pulse Sequence	s2pul	Solvent	acetone	Sweep Width (Hz)	78125.00	Temperature (degree C)	26.000		

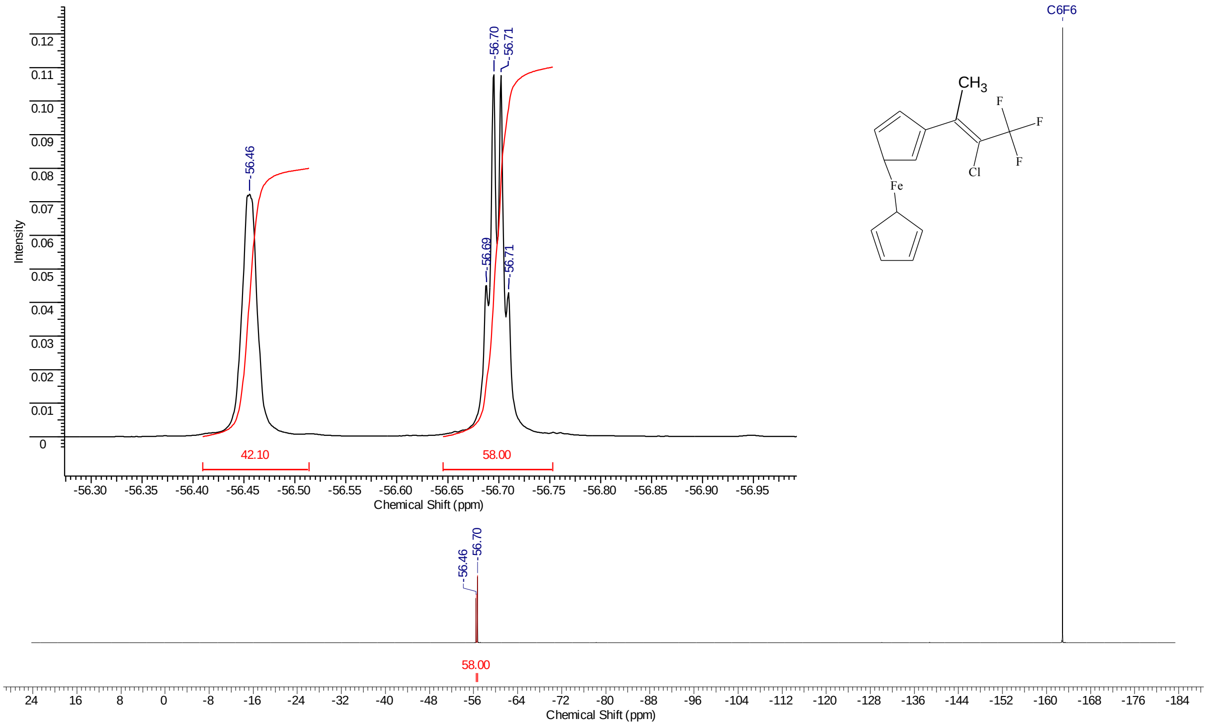

^{19}F NMR spectrum of **9** (376.3 MHz, acetone-d_6)

3 Jul 2015

Acquisition Time (sec)	2.5559	Comment	Imported from UXNMR.			Date	30 Jun 2015 18:25:14
File Name	D:\BN\Docs (BN)\vasiliy\Manusr\Belstein_Ferrocene\SPEC_Fcd\BM-683.H_001001r					Frequency (MHz)	400.13
Nucleus	1H	Number of Transients	4	Original Points Count	16384	Points Count	65536
Pulse Sequence	zg30	Solvent	Acetone	Sweep Width (Hz)	6410.26	Temperature (degree C)	27.000

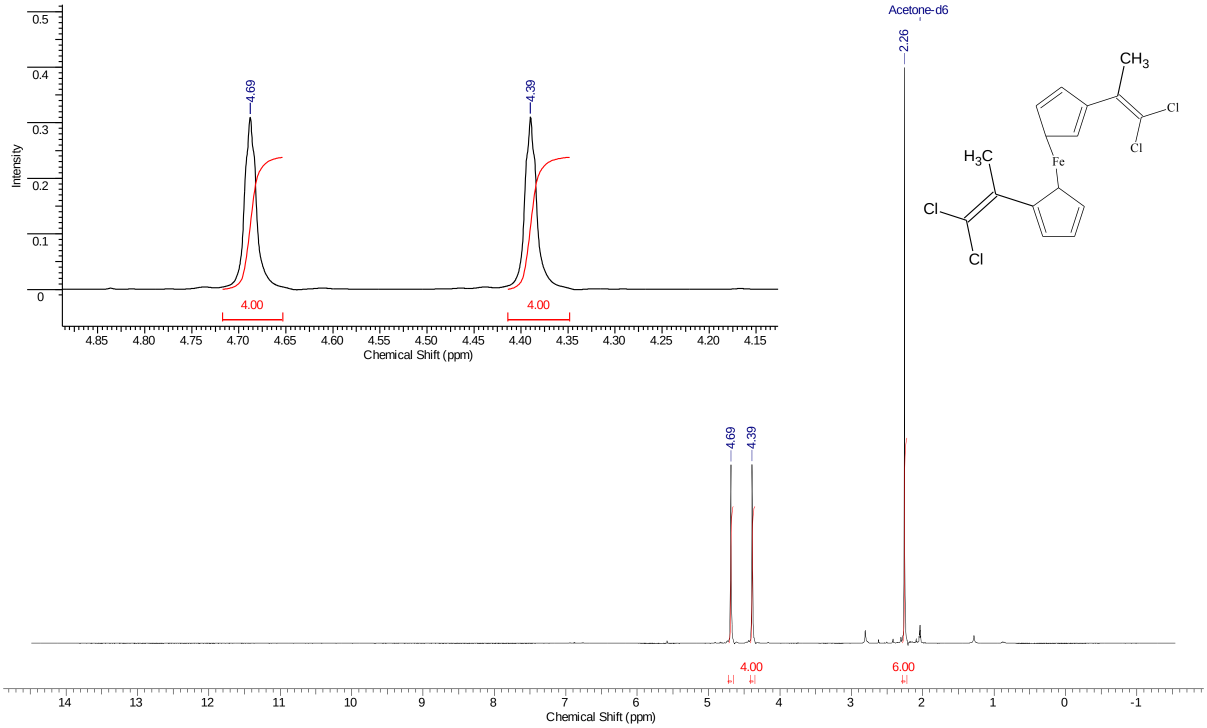

^{1}H NMR spectrum of **10** (400.1 MHz, $CDCl_3$)

3 Jul 2015

Acquisition Time (sec)	0.4999	Comment	Imported from UXNMR.			Date	30 Jun 2015 18:29:04
File Name	D:\BN\Docs (BN)\vasiliy\Manusr\Belstein_Ferrocene\SPEC_Fcd\BM-683.C_002001r					Frequency (MHz)	100.61
Nucleus	13C	Number of Transients	64	Original Points Count	12076	Points Count	65536
Pulse Sequence	zgpg30	Solvent	DMSO-D6	Sweep Width (Hz)	24154.59	Temperature (degree C)	27.000

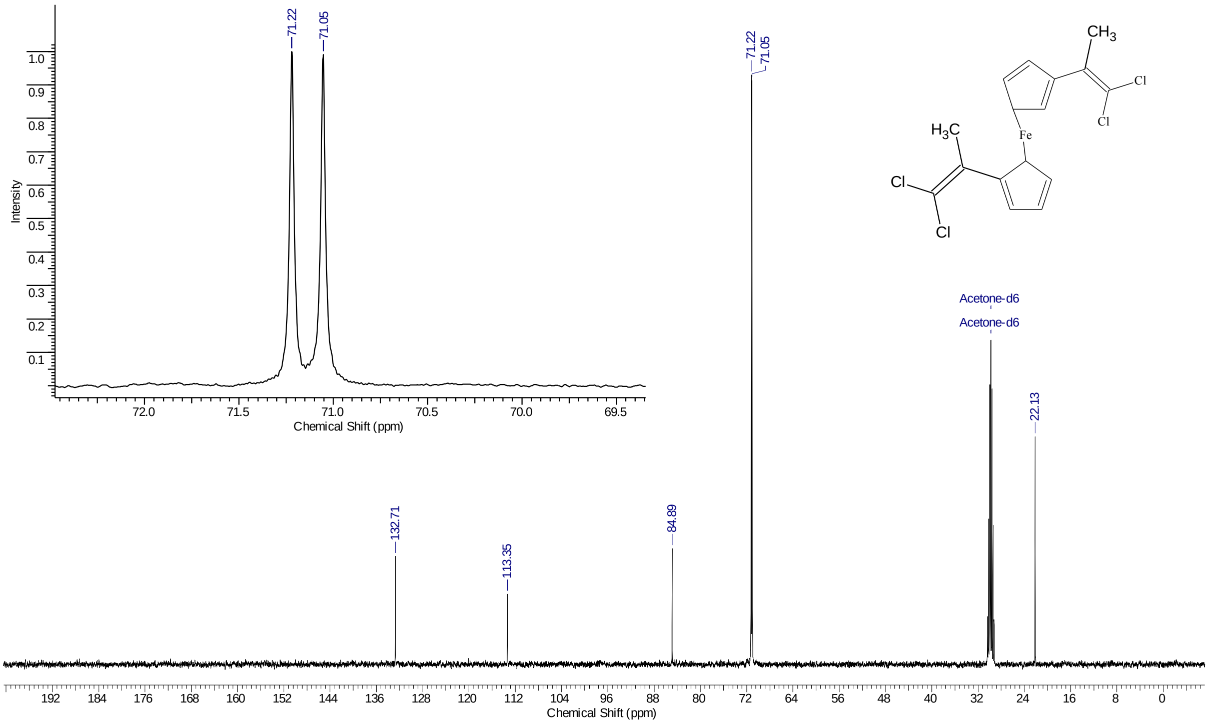

^{13}C NMR spectrum of **10** (100.6 MHz, $CDCl_3$)

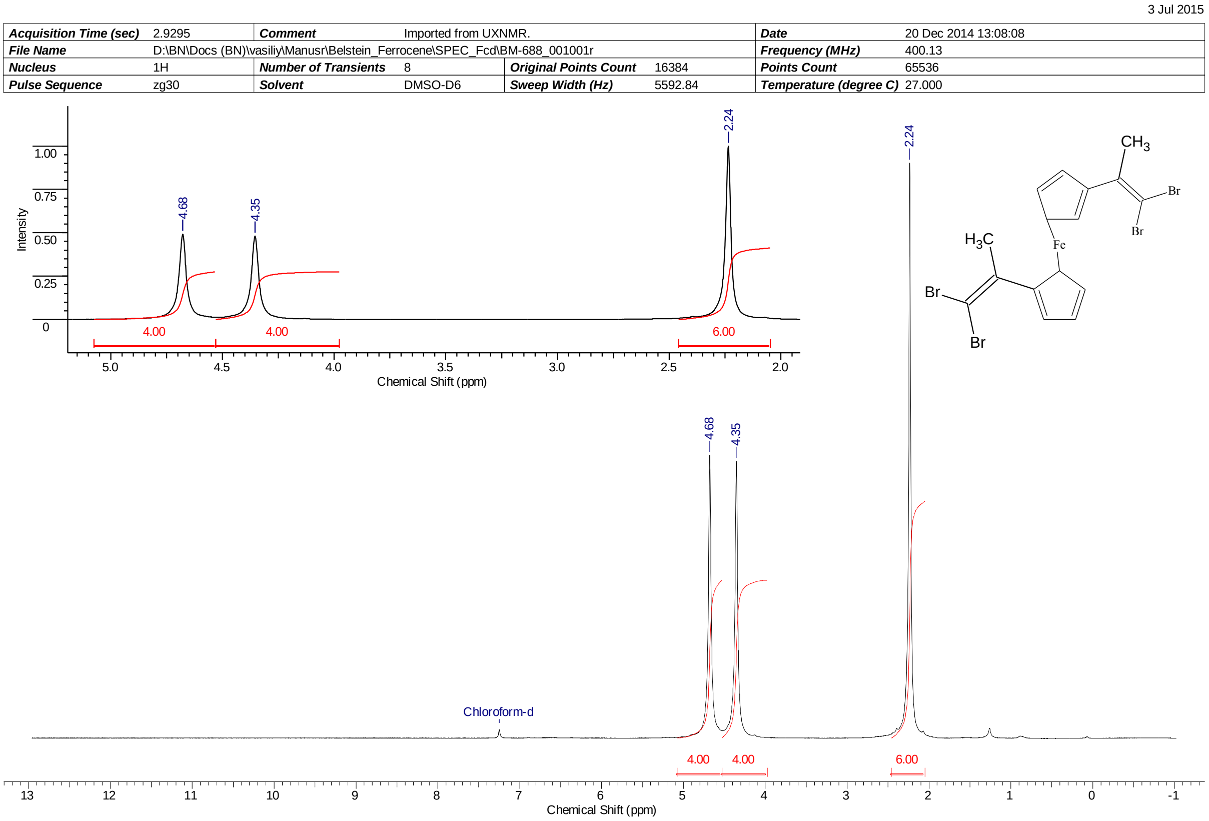

^{1}H NMR spectrum of **11**(400.1 MHz, $CDCl_3$)

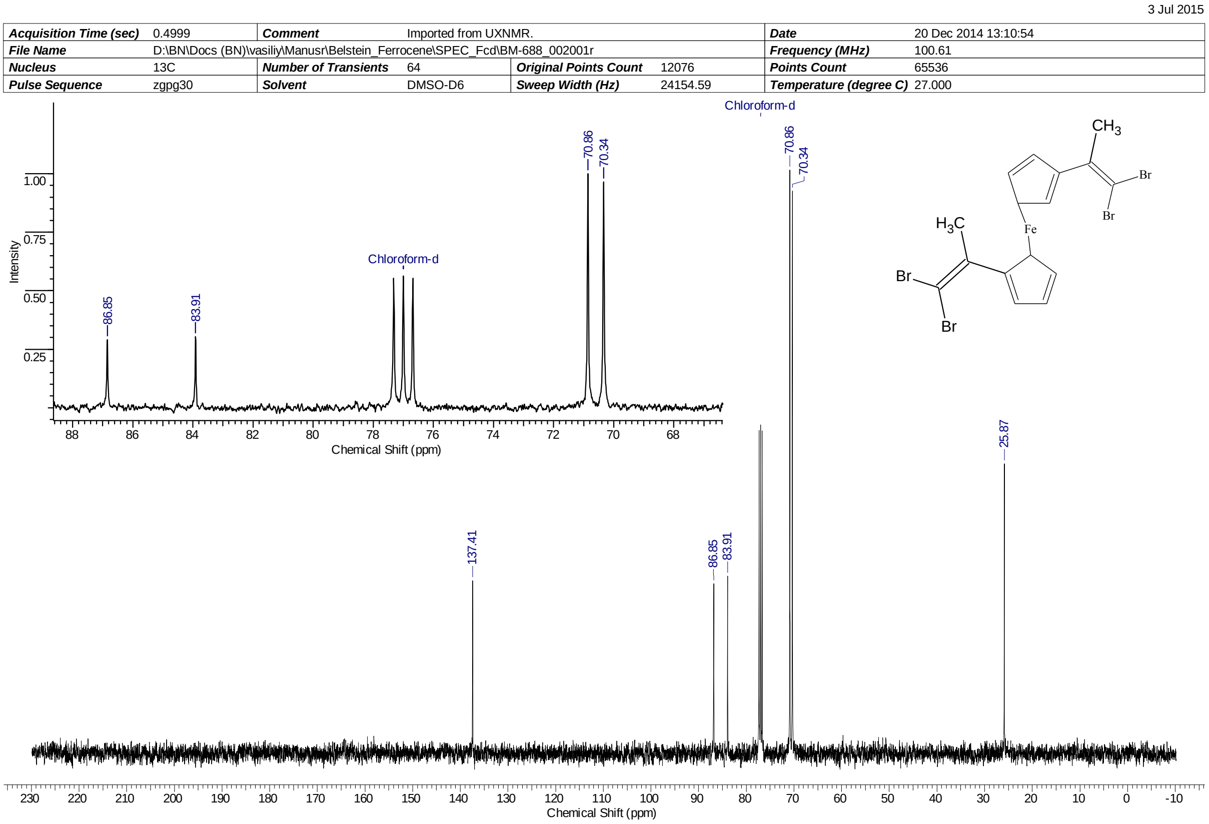

^{13}C NMR spectrum of **11** (100.6 MHz, $CDCl_3$)

4 Jul 2015

Acquisition Time (sec)	2.9295	***Comment***	Imported from UXNMR.			***Date***	20 Dec 2014 13:29:52
File Name	D:\BN\Docs (BN)\vasiliy\Manusr\Belstein_Ferrocene\SPEC_Fcd\BM-690_001001r					***Frequency (MHz)***	400.13
Nucleus	1H	***Number of Transients***	8	***Original Points Count***	16384	***Points Count***	65536
Pulse Sequence	zg30	***Solvent***	DMSO-D6	***Sweep Width (Hz)***	5592.84	***Temperature (degree C)***	27.000

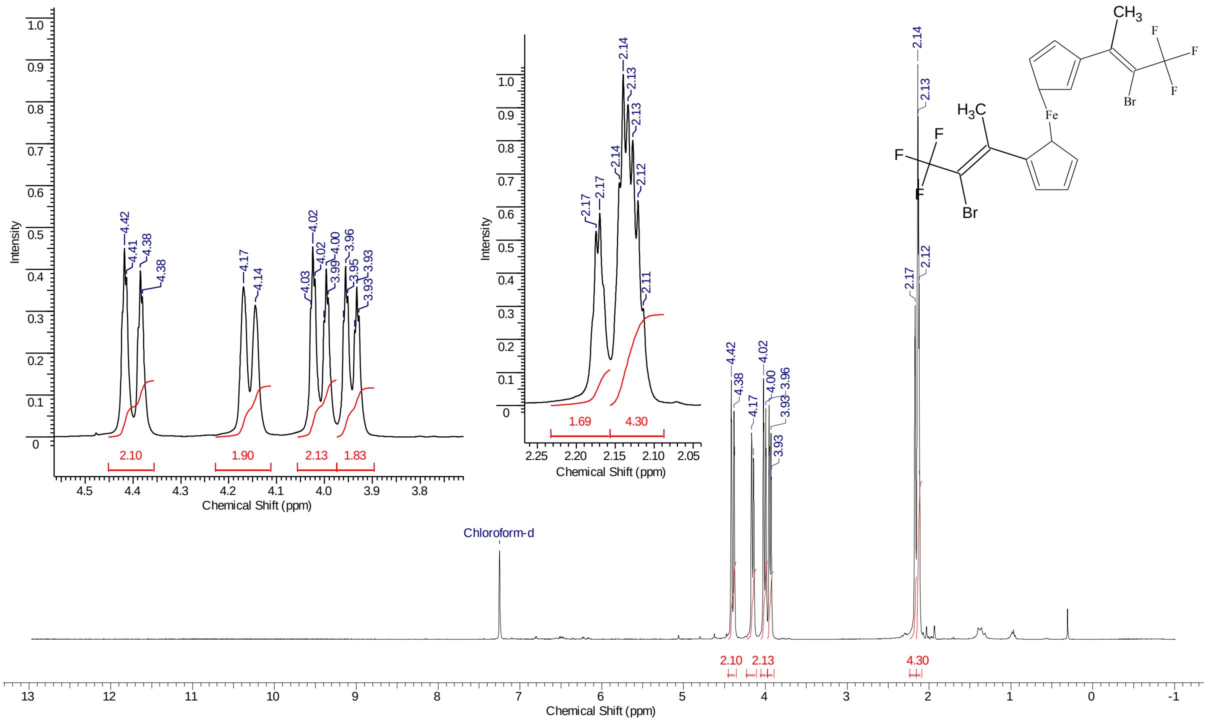

^{1}H NMR spectrum of **12** (400.1 MHz, C_6D_6)

4 Jul 2015

Acquisition Time (sec)	0.4999	***Comment***	Imported from UXNMR.			***Date***	20 Dec 2014 13:37:26
File Name	D:\BN\Docs (BN)\vasiliy\Manusr\Belstein_Ferrocene\SPEC_Fcd\BM-690_002001r					***Frequency (MHz)***	100.61
Nucleus	13C	***Number of Transients***	256	***Original Points Count***	12076	***Points Count***	65536
Pulse Sequence	zgpg30	***Solvent***	DMSO-D6	***Sweep Width (Hz)***	24154.59	***Temperature (degree C)***	27.000

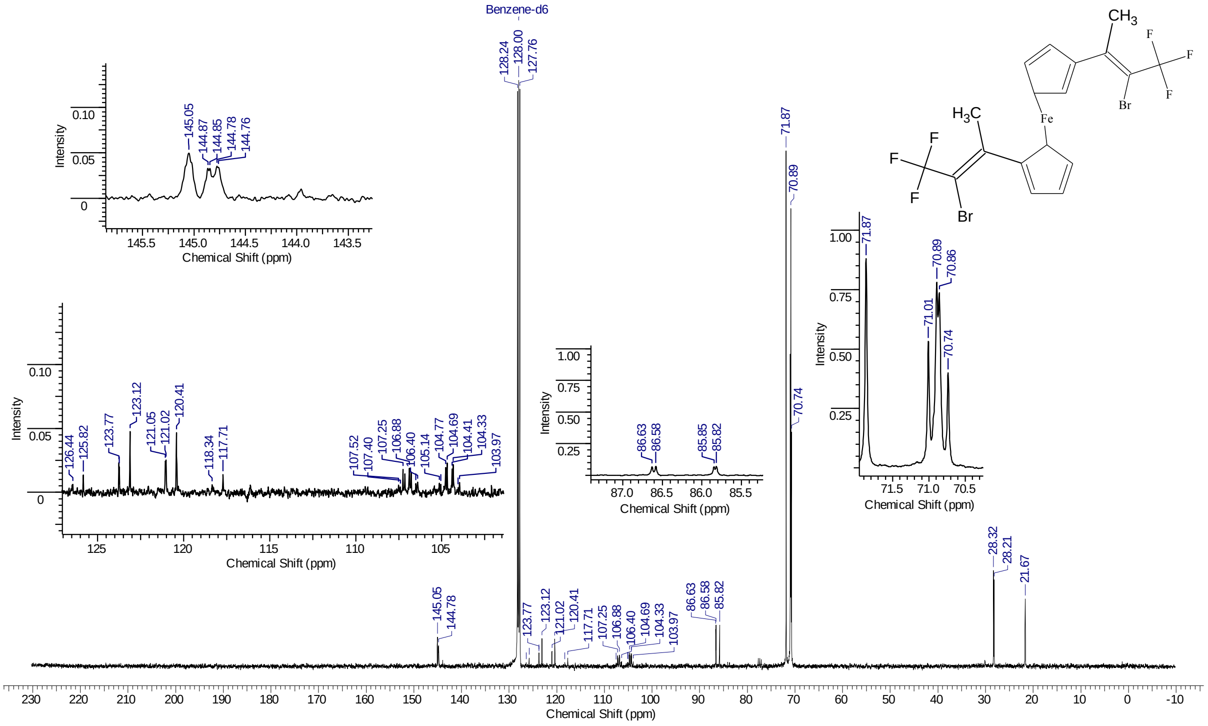

^{13}C NMR spectrum of **12** (100.6 MHz, C_6D_6)

4 Jul 2015

Acquisition Time (sec)	2.0000	Date	Dec 15 2014				
File Name	D:\BN\Docs (BN)\vasiliy\Manusr\Belstein_Ferrocene\SPEC_Fcd\19F\BM-690_20141215_01\FLUORINE_01					Frequency (MHz)	376.31
Nucleus	19F	Number of Transients	256	Original Points Count	178571	Points Count	262144
Pulse Sequence	s2pul	Solvent	BENZENE-D6	Sweep Width (Hz)	89285.71	Temperature (degree C)	40.000

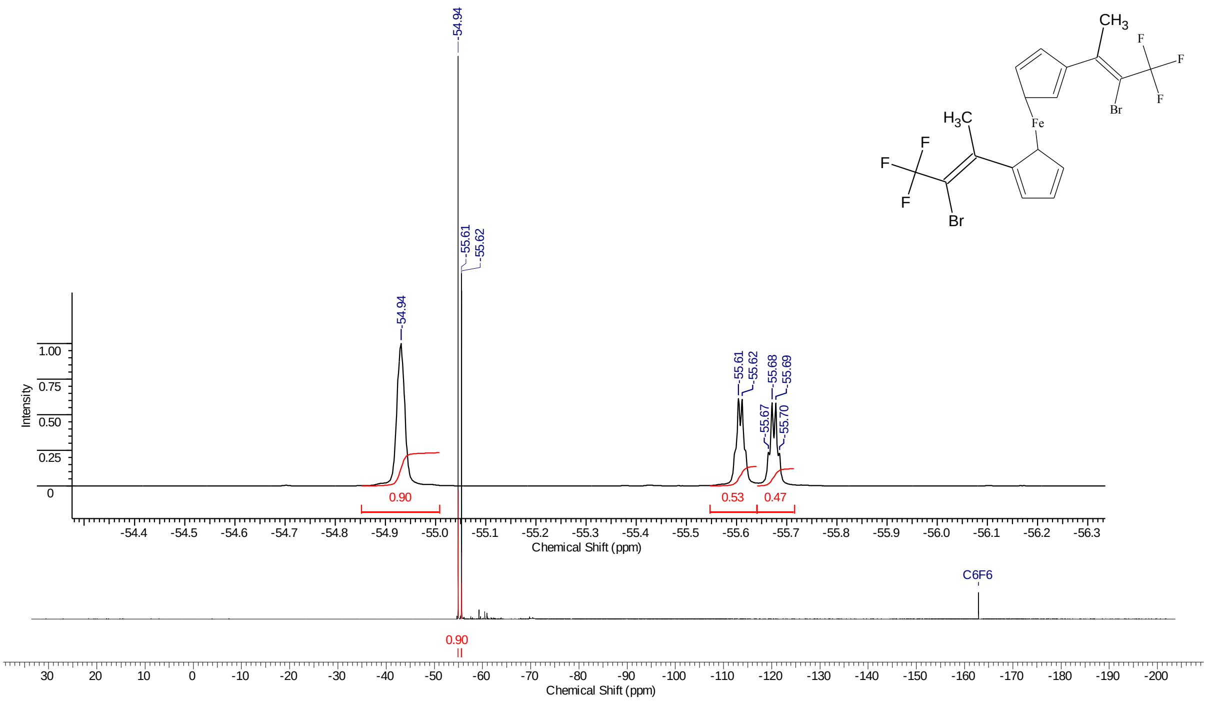

^{19}F NMR spectrum of **12** (376.3 MHz, C_6D_6)

X-ray structure determination. [7] Data were collected on a Bruker APEX-II CCD diffractometer (λ(Mo Kα)-radiation, graphite monochromator, ω and φ scan mode) and corrected for absorption using the SADABS program [8]. For details, see Table S1. The structure was solved by direct methods and refined by full-matrix least squares technique on F^2 with anisotropic displacement parameters for non-hydrogen atoms. The Br and CF_3 substituents are disordered over two same sites with the occupancies of 0.9(*E*-isomer):0.1(*Z*-isomer). The hydrogen atoms were placed in calculated positions and refined within the riding model with fixed isotropic displacement parameters (U_{iso}(H) = 1.5U_{eq}(C) for the CH_3-group and U_{iso}(H) = 1.2U_{eq}(C) for the other groups). All calculations were carried out using the SHELXTL program [9]. Crystallographic data for **8** have been deposited with the Cambridge Crystallographic Data Center. CCDC 905424 contains the supplementary crystallographic data for this paper. These data can be obtained free of charge from the Director, CCDC, 12 Union Road, Cambridge CB2 1EZ, UK (Fax: +44 1223 336033; email: deposit@ccdc.cam.ac.uk or www.ccdc.cam.ac.uk).

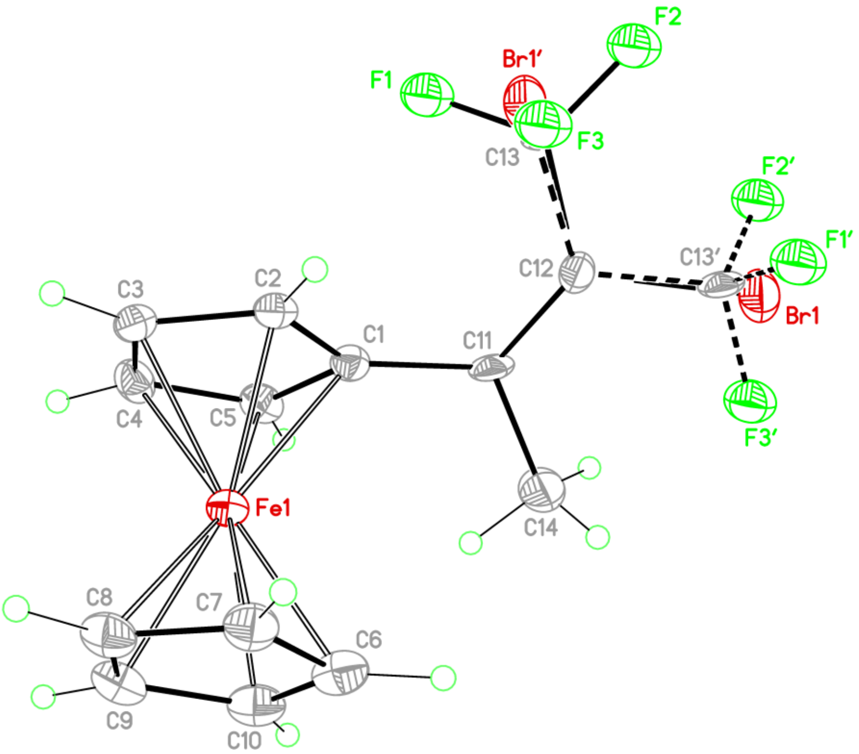

Figure S1: Molecular structure of **8** (50% ellipsoids). The minor ***Z*** isomer is depicted by dashed lines.

Table S1: Crystallographic data for **8.**

Compound	**8**
Empirical formula	$C_{14}H_{12}BrF_3Fe$
Fw	373.00
T, K	100(2)
Crystal size, mm	0.30 x 0.20 x 0.05
Crystal system	Monoclinic
Space group	$P2_1/c$
a, Å	7.6257(4)
b, Å	8.5982(5)
c, Å	19.6528(11)
α, deg.	90
β, deg.	93.128(1)
γ, deg.	90
V, Å^3	1286.66(12)
Z	4
d_c, g · cm^{-3}	1.926
$F(000)$	736
μ, mm^{-1}	4.294
$2\theta_{max}$, deg.	58
Index range	$-10 <= h <= 10$
	$-11 <= k <= 11$
	$-26 <= l <= 26$
No. of rflns collected	15214
No. of unique rflns	3407 (R_{int} = 0.0470)
No. of rflns with $I > 2\sigma(I)$	2663
Data/restraints/parameters	3407 / 38 / 170
$R1$; $wR2$ ($I > 2\sigma(I)$)	0.0545; 0.1369
$R1$; $wR2$ (all data)	0.0722; 0.1456
GOF on F^2	1.002

T_{min}; T_{max}	0.350; 0.800

Table S2: Atomic coordinates (x 10^4) and equivalent isotropic displacement parameters (Å^2 x 10^3) For **8**. U(eq) is defined as one third of the trace of the orthogonalized U^{ij} tensor.

Atom	x	y	z	U(eq)
Br(1)	3703(1)	2546(1)	-1037(1)	27(1)
Br(1')	6824(2)	2577(2)	128(2)	27(1)
Fe(1)	2333(1)	1691(1)	1839(1)	16(1)
F(1)	6899(2)	3458(2)	576(1)	28(1)
F(2)	7192(2)	3174(2)	-495(1)	28(1)
F(3)	6903(2)	1164(2)	138(1)	28(1)
F(1')	4550(4)	1279(2)	-1107(1)	28(1)
F(2')	4616(4)	3780(2)	-1177(1)	28(1)
F(3')	2197(2)	2612(4)	-976(2)	28(1)
C(1)	3549(1)	2961(1)	1114(1)	17(1)
C(2)	4784(1)	1990(1)	1493(1)	18(1)
C(3)	4775(1)	2418(1)	2197(1)	20(1)
C(4)	3536(2)	3645(2)	2244(2)	21(1)
C(5)	2792(2)	3972(2)	1581(1)	20(1)
C(6)	513(4)	207(4)	1385(2)	26(1)
C(7)	1779(4)	-639(4)	1805(2)	23(1)
C(8)	1708(4)	-72(4)	2483(2)	22(1)
C(9)	420(4)	1126(4)	2483(2)	24(1)
C(10)	-324(4)	1291(4)	1805(2)	25(1)
C(11)	3196(1)	2853(1)	378(1)	19(1)
C(12)	4389(1)	2693(1)	-94(1)	17(1)
C(13)	6325(2)	2606(1)	35(1)	19(1)
C(13')	3934(3)	2590(2)	-842(1)	19(1)
C(14)	1271(3)	2944(1)	148(2)	30(1)

Table S3: Bond lengths [Å] and angles [°] for **8**.

Br(1)-C(12)	1.9033(11)	C(3)-C(4)	1.422(2)
Br(1')-C(12)	1.8867(18)	C(3)-H(3)	1.0000
Fe(1)-C(2)	2.0396(12)	C(4)-C(5)	1.419(4)
Fe(1)-C(9)	2.041(3)	C(4)-H(4)	1.0000
Fe(1)-C(8)	2.047(3)	C(5)-H(5)	1.0000
Fe(1)-C(7)	2.048(3)	C(6)-C(10)	1.419(5)
Fe(1)-C(3)	2.0517(13)	C(6)-C(7)	1.434(5)
Fe(1)-C(10)	2.053(3)	C(6)-H(6)	1.0000
Fe(1)-C(6)	2.053(3)	C(7)-C(8)	1.422(5)
Fe(1)-C(4)	2.054(2)	C(7)-H(7)	1.0000
Fe(1)-C(1)	2.0566(14)	C(8)-C(9)	1.424(5)
Fe(1)-C(5)	2.0604(17)	C(8)-H(8)	1.0000
F(1)-C(13)	1.3449(19)	C(9)-C(10)	1.426(5)
F(2)-C(13)	1.3548(18)	C(9)-H(9)	1.0000
F(3)-C(13)	1.3276(16)	C(10)-H(10)	1.0000
F(1')-C(13')	1.3372(19)	C(11)-C(12)	1.3401(16)
F(2')-C(13')	1.3378(19)	C(11)-C(14)	1.514(2)
F(3')-C(13')	1.336(2)	C(12)-C(13)	1.4863(16)
C(1)-C(5)	1.412(2)	C(12)-C(13')	1.4950(17)
C(1)-C(2)	1.4360(16)	C(14)-H(14A)	0.9800
C(1)-C(11)	1.460(2)	C(14)-H(14B)	0.9800
C(2)-C(3)	1.432(3)	C(14)-H(14C)	0.9800
C(2)-H(2)	1.0000		
C(2)-Fe(1)-C(9)	159.35(11)	C(3)-Fe(1)-C(10)	159.72(12)
C(2)-Fe(1)-C(8)	123.23(10)	C(2)-Fe(1)-C(6)	122.91(12)
C(9)-Fe(1)-C(8)	40.76(13)	C(9)-Fe(1)-C(6)	68.44(14)
C(2)-Fe(1)-C(7)	107.69(10)	C(8)-Fe(1)-C(6)	68.54(14)
C(9)-Fe(1)-C(7)	68.55(14)	C(7)-Fe(1)-C(6)	40.95(13)
C(8)-Fe(1)-C(7)	40.64(13)	C(3)-Fe(1)-C(6)	156.62(11)
C(2)-Fe(1)-C(3)	40.97(9)	C(10)-Fe(1)-C(6)	40.44(14)
C(9)-Fe(1)-C(3)	121.76(12)	C(2)-Fe(1)-C(4)	68.15(8)
C(8)-Fe(1)-C(3)	104.58(11)	C(9)-Fe(1)-C(4)	105.91(12)
C(7)-Fe(1)-C(3)	119.44(10)	C(8)-Fe(1)-C(4)	118.74(12)
C(2)-Fe(1)-C(10)	158.59(12)	C(7)-Fe(1)-C(4)	154.19(12)
C(9)-Fe(1)-C(10)	40.77(14)	C(3)-Fe(1)-C(4)	40.53(7)
C(8)-Fe(1)-C(10)	68.50(14)	C(10)-Fe(1)-C(4)	124.68(12)
C(7)-Fe(1)-C(10)	68.49(14)	C(6)-Fe(1)-C(4)	162.49(12)

C(2)-Fe(1)-C(1)	41.04(5)	Fe(1)-C(4)-H(4)	125.6
C(9)-Fe(1)-C(1)	157.00(10)	C(1)-C(5)-C(4)	108.53(17)
C(8)-Fe(1)-C(1)	161.91(10)	C(1)-C(5)-Fe(1)	69.80(8)
C(7)-Fe(1)-C(1)	126.65(10)	C(4)-C(5)-Fe(1)	69.57(10)
C(3)-Fe(1)-C(1)	68.93(7)	C(1)-C(5)-H(5)	125.7
C(10)-Fe(1)-C(1)	123.22(11)	C(4)-C(5)-H(5)	125.7
C(6)-Fe(1)-C(1)	110.37(11)	Fe(1)-C(5)-H(5)	125.7
C(4)-Fe(1)-C(1)	67.97(9)	C(10)-C(6)-C(7)	107.9(3)
C(2)-Fe(1)-C(5)	68.11(5)	C(10)-C(6)-Fe(1)	69.8(2)
C(9)-Fe(1)-C(5)	121.02(11)	C(7)-C(6)-Fe(1)	69.31(19)
C(8)-Fe(1)-C(5)	154.83(12)	C(10)-C(6)-H(6)	126.0
C(7)-Fe(1)-C(5)	163.90(12)	C(7)-C(6)-H(6)	126.0
C(3)-Fe(1)-C(5)	68.34(7)	Fe(1)-C(6)-H(6)	126.0
C(10)-Fe(1)-C(5)	109.38(11)	C(8)-C(7)-C(6)	107.9(3)
C(6)-Fe(1)-C(5)	127.17(12)	C(8)-C(7)-Fe(1)	69.66(18)
C(4)-Fe(1)-C(5)	40.35(10)	C(6)-C(7)-Fe(1)	69.74(19)
C(1)-Fe(1)-C(5)	40.10(6)	C(8)-C(7)-H(7)	126.1
C(5)-C(1)-C(2)	107.46(16)	C(6)-C(7)-H(7)	126.1
C(5)-C(1)-C(11)	128.65(12)	Fe(1)-C(7)-H(7)	126.1
C(2)-C(1)-C(11)	123.89(11)	C(7)-C(8)-C(9)	108.0(3)
C(5)-C(1)-Fe(1)	70.09(9)	C(7)-C(8)-Fe(1)	69.69(19)
C(2)-C(1)-Fe(1)	68.84(7)	C(9)-C(8)-Fe(1)	69.40(19)
C(11)-C(1)-Fe(1)	125.98(5)	C(7)-C(8)-H(8)	126.0
C(3)-C(2)-C(1)	108.31(12)	C(9)-C(8)-H(8)	126.0
C(3)-C(2)-Fe(1)	69.97(7)	Fe(1)-C(8)-H(8)	126.0
C(1)-C(2)-Fe(1)	70.11(6)	C(8)-C(9)-C(10)	108.1(3)
C(3)-C(2)-H(2)	125.8	C(8)-C(9)-Fe(1)	69.84(19)
C(1)-C(2)-H(2)	125.8	C(10)-C(9)-Fe(1)	70.05(19)
Fe(1)-C(2)-H(2)	125.8	C(8)-C(9)-H(9)	125.9
C(4)-C(3)-C(2)	106.95(18)	C(10)-C(9)-H(9)	125.9
C(4)-C(3)-Fe(1)	69.80(9)	Fe(1)-C(9)-H(9)	125.9
C(2)-C(3)-Fe(1)	69.06(8)	C(6)-C(10)-C(9)	108.1(3)
C(4)-C(3)-H(3)	126.5	C(6)-C(10)-Fe(1)	69.80(19)
C(2)-C(3)-H(3)	126.5	C(9)-C(10)-Fe(1)	69.18(19)
Fe(1)-C(3)-H(3)	126.5	C(6)-C(10)-H(10)	126.0
C(5)-C(4)-C(3)	108.8(2)	C(9)-C(10)-H(10)	126.0
C(5)-C(4)-Fe(1)	70.08(11)	Fe(1)-C(10)-H(10)	126.0
C(3)-C(4)-Fe(1)	69.66(10)	C(12)-C(11)-C(1)	126.57(9)
C(5)-C(4)-H(4)	125.6	C(12)-C(11)-C(14)	118.88(16)
C(3)-C(4)-H(4)	125.6	C(1)-C(11)-C(14)	114.55(15)

C(11)-C(12)-C(13)	126.36(11)	F(3')-C(13')-F(1')	107.49(19)
C(11)-C(12)-C(13')	123.79(12)	F(3')-C(13')-F(2')	107.48(19)
C(11)-C(12)-Br(1')	122.85(12)	F(1')-C(13')-F(2')	107.40(18)
C(13')-C(12)-Br(1')	113.37(14)	F(3')-C(13')-C(12)	111.51(18)
C(11)-C(12)-Br(1)	121.29(9)	F(1')-C(13')-C(12)	111.40(16)
C(13)-C(12)-Br(1)	112.34(10)	F(2')-C(13')-C(12)	111.34(15)
F(3)-C(13)-F(1)	107.31(15)	C(11)-C(14)-H(14A)	109.5
F(3)-C(13)-F(2)	106.37(14)	C(11)-C(14)-H(14B)	109.5
F(1)-C(13)-F(2)	105.05(13)	H(14A)-C(14)-H(14B)	109.5
F(3)-C(13)-C(12)	113.08(11)	C(11)-C(14)-H(14C)	109.5
F(1)-C(13)-C(12)	112.62(13)	H(14A)-C(14)-H(14C)	109.5
F(2)-C(13)-C(12)	111.87(13)	H(14B)-C(14)-H(14C)	109.5

Table S4: Anisotropic displacement parameters ($Å^2 \times 10^3$) for **8**. The anisotropic displacement factor exponent takes the form: $-2\pi^2[h^2 a^{*2}U^{11} + ... + 2 h k a^* b^* U^{12}]$

Atom	U^{11}	U^{22}	U^{33}	U^{23}	U^{13}	U^{12}
Br(1)	29(1)	35(1)	18(1)	-2(1)	0(1)	2(1)
Br(1')	29(1)	35(1)	18(1)	-2(1)	0(1)	2(1)
Fe(1)	15(1)	15(1)	18(1)	1(1)	2(1)	-1(1)
F(1)	20(1)	36(1)	29(1)	3(1)	3(1)	-1(1)
F(2)	20(1)	36(1)	29(1)	3(1)	3(1)	-1(1)
F(3)	20(1)	36(1)	29(1)	3(1)	3(1)	-1(1)
F(1')	20(1)	36(1)	29(1)	3(1)	3(1)	-1(1)
F(2')	20(1)	36(1)	29(1)	3(1)	3(1)	-1(1)
F(3')	20(1)	36(1)	29(1)	3(1)	3(1)	-1(1)
C(1)	16(1)	17(1)	17(1)	2(1)	1(1)	-2(1)
C(2)	14(1)	22(1)	20(1)	1(1)	2(1)	-2(1)
C(3)	15(1)	24(2)	20(1)	2(1)	-1(1)	-4(1)
C(4)	27(2)	19(1)	18(1)	1(1)	5(1)	-5(1)
C(5)	21(1)	17(1)	22(1)	-1(1)	5(1)	-1(1)
C(6)	21(1)	24(2)	32(2)	1(1)	-1(1)	-4(1)
C(7)	23(1)	19(1)	28(2)	0(1)	3(1)	-1(1)
C(8)	23(1)	16(1)	29(2)	6(1)	4(1)	-1(1)
C(9)	23(1)	21(1)	30(2)	2(1)	10(1)	0(1)
C(10)	18(1)	22(1)	36(2)	3(1)	3(1)	0(1)
C(11)	6(1)	27(1)	23(1)	3(1)	-2(1)	-2(1)
C(12)	18(1)	19(1)	14(1)	-1(1)	-2(1)	-1(1)
C(13)	6(1)	27(1)	23(1)	3(1)	-2(1)	-2(1)
C(13')	6(1)	27(1)	23(1)	3(1)	-2(1)	-2(1)
C(14)	17(1)	49(2)	24(2)	3(2)	0(1)	3(2)

Table S5: Hydrogen coordinates (x 10^4) and isotropic displacement parameters ($Å^2$ x 10^3) for **8**.

Atom	x	y	z	U(iso)
H(2)	5526	1160	1298	22
H(3)	5507	1950	2581	24
H(4)	3229	4179	2673	25
H(5)	1871	4772	1467	24
H(6)	270	66	883	31
H(7)	2571	-1478	1649	28
H(8)	2444	-442	2887	27
H(9)	90	1742	2888	29
H(10)	-1259	2048	1651	30
H(14A)	1091	3795	-180	45
H(14B)	572	3134	543	45
H(14C)	905	1960	-68	45

Table S6: Torsion angles [°] for **8**.

C(5)-C(1)-C(2)-C(3)	-0.07(6)	C(2)-C(1)-C(11)-C(14)	136.80(7)
C(11)-C(1)-C(2)-C(3)	-179.70(5)	Fe(1)-C(1)-C(11)-C(14)	49.64(7)
Fe(1)-C(1)-C(2)-C(3)	-59.77(6)	C(1)-C(11)-C(12)-C(13)	-0.55(6)
C(5)-C(1)-C(2)-Fe(1)	59.71(7)	C(14)-C(11)-C(12)-C(13)	179.16(7)
C(11)-C(1)-C(2)-Fe(1)	-119.93(6)	C(1)-C(11)-C(12)-C(13')	-179.99(6)
C(1)-C(2)-C(3)-C(4)	0.06(6)	C(14)-C(11)-C(12)-C(13')	-0.28(7)
Fe(1)-C(2)-C(3)-C(4)	-59.80(6)	C(1)-C(11)-C(12)-Br(1')	0.00(5)
C(1)-C(2)-C(3)-Fe(1)	59.86(5)	C(14)-C(11)-C(12)-Br(1')	179.71(7)
C(2)-C(3)-C(4)-C(5)	-0.04(10)	C(1)-C(11)-C(12)-Br(1)	179.28(3)
Fe(1)-C(3)-C(4)-C(5)	-59.36(9)	C(14)-C(11)-C(12)-Br(1)	-1.01(5)
C(2)-C(3)-C(4)-Fe(1)	59.33(6)	C(11)-C(12)-C(13)-F(3)	89.27(15)
C(2)-C(1)-C(5)-C(4)	0.04(10)	Br(1)-C(12)-C(13)-F(3)	-90.58(15)
C(11)-C(1)-C(5)-C(4)	179.66(8)	C(11)-C(12)-C(13)-F(1)	-32.60(14)
Fe(1)-C(1)-C(5)-C(4)	58.96(9)	Br(1)-C(12)-C(13)-F(1)	147.56(11)
C(2)-C(1)-C(5)-Fe(1)	-58.92(5)	C(11)-C(12)-C(13)-F(2)	-150.65(11)
C(11)-C(1)-C(5)-Fe(1)	120.70(7)	Br(1)-C(12)-C(13)-F(2)	29.50(13)
C(3)-C(4)-C(5)-C(1)	0.00(12)	C(11)-C(12)-C(13')-F(3')	-2.6(2)
Fe(1)-C(4)-C(5)-C(1)	-59.11(8)	Br(1')-C(12)-C(13')-F(3')	177.41(18)
C(3)-C(4)-C(5)-Fe(1)	59.10(8)	C(11)-C(12)-C(13')-F(1')	-122.70(16)
C(10)-C(6)-C(7)-C(8)	0.2(4)	Br(1')-C(12)-C(13')-F(1')	57.31(18)
Fe(1)-C(6)-C(7)-C(8)	59.5(2)	C(11)-C(12)-C(13')-F(2')	117.43(17)
C(10)-C(6)-C(7)-Fe(1)	-59.3(2)	Br(1')-C(12)-C(13')-F(2')	-62.56(18)
C(6)-C(7)-C(8)-C(9)	-0.5(4)		
Fe(1)-C(7)-C(8)-C(9)	59.0(2)		
C(6)-C(7)-C(8)-Fe(1)	-59.5(2)		
C(7)-C(8)-C(9)-C(10)	0.6(4)		
Fe(1)-C(8)-C(9)-C(10)	59.8(2)		
C(7)-C(8)-C(9)-Fe(1)	-59.2(2)		
C(7)-C(6)-C(10)-C(9)	0.2(4)		
Fe(1)-C(6)-C(10)-C(9)	-58.8(2)		
C(7)-C(6)-C(10)-Fe(1)	59.0(2)		
C(8)-C(9)-C(10)-C(6)	-0.5(4)		
Fe(1)-C(9)-C(10)-C(6)	59.2(2)		
C(8)-C(9)-C(10)-Fe(1)	-59.7(2)		
C(5)-C(1)-C(11)-C(12)	136.97(8)		
C(2)-C(1)-C(11)-C(12)	-43.48(7)		
Fe(1)-C(1)-C(11)-C(12)	-130.63(5)		
C(5)-C(1)-C(11)-C(14)	-42.75(9)		

References

1. Broadhead, G. D.; Osgerby, J. M.; Pauson, P. L. *J. Chem. Soc* **1958**, 650-656. DOI: 10.1039/JR9580000650.

2. Graham, P.J.; Lindsey, R.V.; Parshall, G.W.; Peterson, M.L.; Whitman, G.M. *J. Am. Chem. Soc.*, **1957**, *79*, 3416-3420. DOI: 10.1021/ja01570a027.

3. Carroll, M. A.; White, A. J. P.; Widdowson, D. A.; Williams, D. J. *J. Chem. Soc., Perkin Trans. 1*, **2000**, 1551-1557. DOI: 10.1039/B000833H.

4. Osborne, W. Da S.; Hursthouse, M.; Opromolla, Z. *J. Organomet. Chem.*, **1996**, *516*, 167–176. DOI:10.1016/0022-328X(96)06138-4.

5. Luo, S. J.; Liu, Y. H.; Liu, C. M.; Liang, Y. M.; Ma, Y. X. *Synth. Commun*., **2000**, *30*, 1569–1572. DOI: 10.1080/00397910008087190.

6. Tsuboya, N.; Hamasaki, R.; Ito, M.; Mitsuishi, M.; Miyashita, T.; Yamamoto, Y. *J. Mater. Chem.*, **2003**, *13*, 511-513. DOI: 10.1039/B211019A.

7. Shixaliyev, N. G.; Heydarova, S. J.; Muzalevskiy, V. M.; Nenaydenko, V. G.; Rahimova, A. G. *Azerb. Khim. Zh.*, **2013**, 78-83.

8. Sheldrick, G. M. SADABS, v. 2.03, Bruker/Siemens Area Detector Absorption Correction Program, Bruker AXS, Madison, Wisconsin, 2003.

9. Sheldrick, G. M. *Acta Cryst. Sect.* A 64, 2008, 112.
